# 1 kHz fixed-target serial crystallography using a multilayer monochromator and an integrating pixel detector

**DOI:** 10.1107/S205225251900914X

**Published:** 2019-08-17

**Authors:** A. Tolstikova, M. Levantino, O. Yefanov, V. Hennicke, P. Fischer, J. Meyer, A. Mozzanica, S. Redford, E. Crosas, N. L. Opara, M. Barthelmess, J. Lieske, D. Oberthuer, E. Wator, I. Mohacsi, M. Wulff, B. Schmitt, H. N. Chapman, A. Meents

**Affiliations:** a Center for Free Electron Laser Science, DESY, Notkestrasse 85, 22607 Hamburg, Germany; b Department of Physics, University of Hamburg, Luruper Chaussee 149, 22761 Hamburg, Germany; c European Synchrotron Radiation Facility, 71 Avenue des Martyrs, 38000 Grenoble, France; d Deutsches Elektronen Synchrotron, Photon Science, Notkestrasse 85, 22607 Hamburg, Germany; e Paul Scherrer Institute, 111 Forschungsstrasse, 5232 Villigen, Switzerland; f C-CINA, Biozentrum, University of Basel, Mattenstrasse 26, 4002 Basel, Switzerland; g Malopolska Centre of Biotechnology, Jagiellonian University, Cracow 30-387, Poland; h Centre for Ultrafast Imaging, University of Hamburg, Luruper Chaussee 149, Hamburg 22761, Germany

**Keywords:** serial crystallography, synchrotron radiation, pink beams, protein crystallography, protein structure, structure determination

## Abstract

Reliable sample delivery and efficient use of limited beam time have remained bottlenecks for serial crystallography (SX). Using a high-intensity polychromatic X-ray beam in combination with a newly developed charge-integrating JUNGFRAU detector, we have applied the method of fixed-target SX to collect data at a rate of 1 kHz at a synchrotron-radiation facility. According to our data analysis for the given experimental conditions, only about 3 000 diffraction patterns are required for a high-quality diffraction dataset. With indexing rates of up to 25%, recording of such a dataset takes less than 30 s.

## Abstract 2

1 kHz fixed-target serial crystallography using polychromatic X-rays and a novel JUNGFRAU detector allows the recording of a complete diffraction dataset in 30 s.

## 1. Introduction   

Serial crystallography (SX) is based on merging data from still diffraction patterns collected from several tens to several hundreds of thousands of crystals into a complete set of structure factors, which is then used for protein structure determination (Chapman *et al.*, 2011 ▸; Schlichting, 2015 ▸). In contrast, conventional macromolecular X-ray crystallography is based on collecting a series of rotation photographs from one or more crystals, which are then merged into a complete dataset. By avoiding the need to rotate crystals over a certain angle increment during their exposure and requiring individual measurements of only minimal signal to background, SX thus allows almost arbitrarily short exposure times, making this method ideally suited for time-resolved experiments.

SX employs a variety of methods to deliver individual microcrystals to the X-ray beam for data collection (Grünbein & Kovacs, 2019 ▸; Martiel *et al.*, 2019 ▸). For fixed-target SX experiments the microcrystals are immobilized on a solid support and systematically raster scanned through the X-ray beam. Fixed-target sample delivery has the advantage of being highly reliable, yields high hit rates and requires only minimal amounts of sample. This approach has thereby removed several bottlenecks for SX experiments at X-ray free-electron lasers (XFELs) and also at synchrotron facilities (Roedig *et al.*, 2015 ▸, 2016 ▸, 2017 ▸; Hunter *et al.*, 2014 ▸).

Fixed-target SX experiments at XFELs, such as the Linac Coherent Light Source in the US, are typically performed using single exposures from femtosecond X-ray pulses, which consequently destroy the sample because of their extremely high intensities of up to 10^12^ photons typically focused into a few micrometre spot. For a pulse duration short enough to outrun radiation damage and a given pulse fluence, the number of patterns required to complete a high-quality dataset of structure factors also depends on factors such as crystal size, symmetry, as well as the bandwidth of the radiation (White *et al.*, 2013 ▸). With a bandwidth of 0.2%, experiments with pulses of these parameters usually require a few thousand still diffraction patterns to complete a high-quality dataset of structure factors.

By removing the requirement to obtain high signal-to-background data from individual crystals and by reducing the dose by spreading the exposure over many crystals, SX is also a compelling method at synchrotron sources. Using available beamlines, several such experiments have been conducted using monochromatic radiation (Stellato *et al.*, 2014 ▸; Beyerlein *et al.*, 2017 ▸). With an X-ray flux of 10^12^–10^13^ photons s^−1^ focused into a 10 × 10 µm spot, exposure times at these instruments are typically in the few milliseconds range, to obtain a sufficient diffraction signal from small crystals. Using a much smaller bandwidth of only 0.01%, SX experiments with monochromatic synchrotron radiation typically require more than tens of thousands of diffraction still images to obtain a complete high-quality dataset (Stellato *et al.*, 2014 ▸).

Recently we demonstrated SX measurements at a synchrotron facility using a polychromatic ‘pink’ X-ray beam with exposure times of 100 ps only (Meents *et al.*, 2017 ▸). Using a full harmonic of the undulator spectrum with a bandwidth of 5.7% [full width at half-maximum (FWHM)], still diffraction patterns from about 50 crystals were sufficient to obtain a high-quality dataset. In contrast to previous SX experiments using radiation of narrower bandwidth, processing of these data turned out to be challenging, with only 13% of patterns being amenable to indexing using available algorithms (Meents *et al.*, 2017 ▸). This difficulty was attributed to the low-energy tail of the polychromatic beam, which extends to photon energies 20% lower than the peak energy, giving rise to the large number of Bragg spots observed in a diffraction pattern. Automatic indexing algorithms used for monochromatic radiation are not suitable for processing such patterns as it is impossible to distinguish which wavelength of the incident polychromatic beam diffracted into a particular spot and hence calculate its reciprocal-space coordinates. Available pink-beam indexing algorithms rely on finding ellipses in the diffraction-spots patterns and often fail when small or weakly diffracting crystals are used which is typically the case in SX experiments. Furthermore, no fully automatic data-processing suite is available for polychromatic single-crystal X-ray diffraction data, as the software requires prior knowledge of the unit-cell parameters and substantial manual input.

It has been recently reported that usable intensities can be obtained from sparse pink-beam diffraction data using the standard pipeline for monochromatic data processing (Martin-Garcia *et al.*, 2019 ▸). A limitation of this approach is that only 10% of the diffraction patterns could be indexed and the valuable high-resolution diffraction spots could not be indexed and were not included in the structure refinement.

Our experience with the indexing of protein crystal diffraction obtained with polychromatic radiation, reported previously (Meents *et al.*, 2017 ▸) and in this article, along with our attempts at indexing simulated diffraction patterns, have shown that existing auto-indexing algorithms tend to fail when the bandwidth increases to the point where there are significant peak overlaps. This is unsurprising given that those algorithms assume monochromatic radiation, but we have found that a good compromise between a high indexing success rate and high beam fluence is obtained with a bandwidth of about 2%, as long as the spectrum is approximately symmetric. Such a spectrum can be obtained at an undulator beamline using a multilayer monochromator, for example. This choice of bandwidth also produces a reasonably high proportion of Bragg peaks that are fully integrated and so accurate structure factors can be estimated from far fewer diffraction patterns than needed when using narrower bandwidth radiation, where most peaks are only partial reflections.

For example, beamline ID09 at the European Synchrotron Radiation Facility (ESRF) provides up to 10^8^ photons in a single 100 ps pulse when using a multilayer monochromator. Higher photon fluxes of more than 10^9^ photons can be achieved with microsecond exposure times. This appears to be an optimal compromise between flux and time resolution for investigating many biological processes.

A challenge for diffraction measurements with a high-flux pink beam is that suitable detectors are rarely available. Hybrid pixel-array photon-counting detectors have limited count rates of several megahertz (Broennimann *et al.*, 2006 ▸). That is, in a 1 µs exposure, it is only possible to count a few photons, at most, in each pixel. Instead, integrating detectors are required. While charge-coupled device (CCD) detectors are an older technology than hybrid photon-counting detectors, with higher noise and slower readout, they are integrating and hence have remained standard detectors for synchrotron experiments using polychromatic X-rays. Recent developments of hybrid charge-integrating pixel detectors, such as the Cornell-SLAC hybrid Pixel Array Detector (CSPAD; Hart *et al.*, 2012 ▸), the Adaptive Gain Integrating Pixel Detector (AGIPD; Allahgholi *et al.*, 2016 ▸) and the adjusting gain detector for the Aramis User station (JUNGFRAU; Mozzanica *et al.*, 2016 ▸), initially developed for XFELs, have also opened new opportunities for high-flux synchrotron experiments as they can handle this high flux and sustain high frame rates (Leonarski *et al.*, 2018 ▸).

In this article, we describe fixed-target SX experiments performed at a frame rate of 1 kHz using a polychromatic synchrotron beam with a bandwidth of 2.5%, using a JUNGFRAU detector and a Roadrunner II goniometer for high-speed sample delivery (Roedig *et al.*, 2017 ▸).

## 2. Experiments   

**Figure 1:**
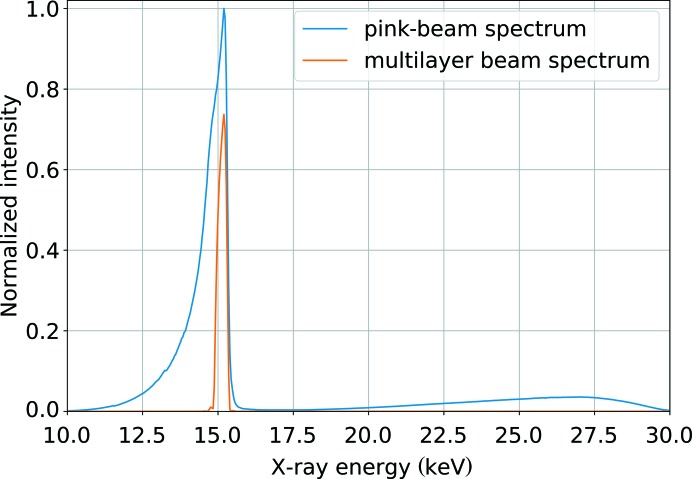
Measured X-ray energy spectrum at beamline ID09 with and without the multilayer monochromator.

Diffraction experiments were performed at beamline ID09 at the ESRF using the multilayer monochromator installed at the instrument. The resulting energy spectrum of the X-rays used for the experiments is shown in Fig. 1 ▸ with an energy spread of 2.5% (FWHM) centered at a photon energy of 15.2 keV. The measured beamsize at the sample position was 60 × 60 µm.

Diffraction patterns were recorded on a JUNGFRAU 1M pixel detector. The detector consisted of two individual 500 K pixel JUNGFRAU modules mounted on top of each other with a vertical gap of 2.8 mm in between them. With the given detector area of 77 × 80 mm and a minimum detector distance of 100 mm limited by geometrical restrictions at the instrument, we decided to offset the detector center horizontally by 26 mm with respect to the incident X-ray beam to allow collection of reflections up to 1.4 Å at the edge of the detector. For 1 kHz data collection, the integration period of the detector was set to 10 µs and the detector was cooled to 18°C during operation.

**Figure 2:**
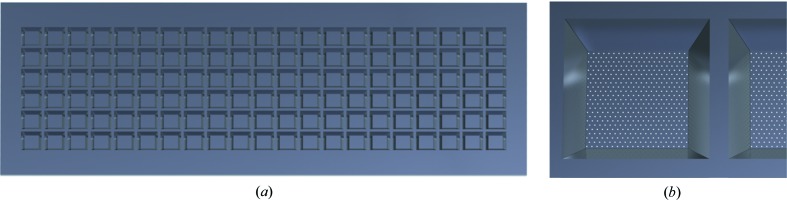
(*a*) Roadrunner II chip with dimensions of 33 × 12 mm (*H* × *V*). The chip provides 21 × 6 compartments each with a size of 1.0 × 1.0 mm separated by a support frame structure with a width of 600 µm and a thickness of 300 µm. (*b*) The membrane thickness of the 126 individual compartments is 10 µm and the membranes are equipped with hexagonal patterns of micropores with diameters of 20 µm and a spacing of 50 µm between the pores. Because of the horizontal beamsize of 60 µm used for the experiments, which is larger than the pore separation, it was decided to expose at intervals of twice the pore spacing in order to avoid double exposure of the same crystal.

Crystals of the two model compounds, lysozyme and proteinase K, were directly grown on micro-patterned silicon chips as described in more detail by Lieske *et al.* (2019 ▸) and in the Supporting information. Crystals of both proteins all had dimensions of about 50 × 50 × 50 µm. A technical drawing of the silicon chips used for the experiment (also referred to as Roadrunner II chips) and the corresponding pore-pattern are shown in Figs. 2 ▸(*a*) and 2 ▸(*b*). For data collection, a Roadrunner chip with crystals was taken out of its crystallization chamber, the crystal-growth solution was removed through the pores by blotting the underside of the chip with filter paper (Roedig *et al.*, 2016 ▸) and the chip was then mounted on the Roadrunner II goniometer installed at the beamline.

**Figure 3:**
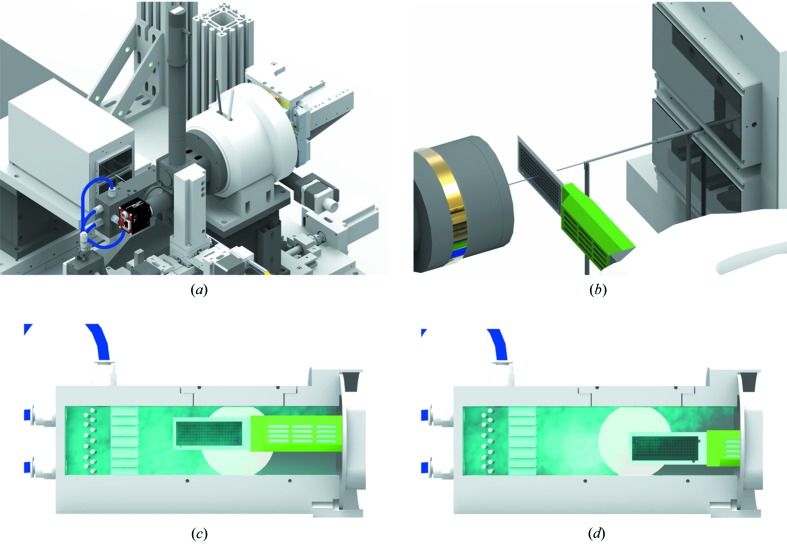
(*a*) Technical drawing of the Roadrunner II goniometer together with the JUNGFRAU 1M detector installed at beamline ID09 at the ESRF. (*b*) Close-up of the interaction region showing the in-line sample-viewing microscope with the collimator (left), the chip with the retracted humidor (in green), the capillary beamstop enclosing the direct beam shortly after the chip and the JUNGFRAU 1M detector (right). For better visibility, the humidity chamber is not shown here. The X-ray beam bath is highlighted in pink. (*c*) The Roadrunner II chip inside the measurement chamber. The observed humidity gradient from the top-left to the lower-right side as observed in the chamber is indicated in light blue, with areas of higher humidity being brighter. In the ‘in-position’ at the start of a scan the whole chip area is in a region of high relative humidity. (*d*) The ‘out-position’ of the chip at the end of a measurement. In particular, the lower-right side of the chip is in an area of lower relative humidity.

In contrast to instrumentation usually available at crystallography endstations, the Roadrunner II goniometer is equipped with a high-speed horizontal scanning stage (*x* axis) capable of scanning at speeds of up to 100 mm s^−1^. This fast scanning axis is mounted on a *y* *z* translation stage allowing for it to be positioned vertically (the *y* direction) and along the X-ray beam direction (the *z* direction). This whole scanning unit can be rotated (by an angle ω) around the *x* axis, using a high-precision air bearing. A technical drawing of the Roadrunner II goniometer, as used for the experiment, is shown in Fig. 3 ▸.

Once mounted onto the scanning unit, each chip was aligned with respect to the X-ray beam with an in-line sample-viewing microscope, and the scanning grid was defined using the *Roadrunner* software. For subsequent data collection, each chip was continuously scanned through the X-ray beam in the horizontal direction with a constant velocity of 100 mm s^−1^. With an X-ray pulse frequency of 1 kHz, generated by an X-ray chopper, this corresponds to a spatial separation of 100 µm between two shots, which is about twice the beamsize at the sample position. During an X-ray exposure of 1 µs, the crystal moves by only 100 nm, which is insignificant compared with the crystal and beam sizes. The scans started at the bottom-right corner of every chip. After a horizontal line scan was finished, each chip was moved down vertically by 100 µm to the next line, rotated by a small ω increment and then scanned along *x* in the reverse direction. This procedure was repeated for the whole chip. More details about the chip alignment and scanning procedure can be found in the work by Roedig *et al.* (2017 ▸).

**Figure 4:**
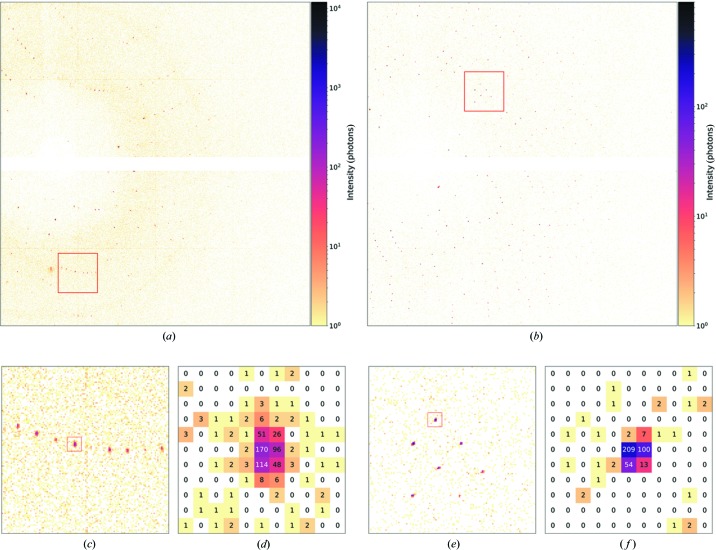
Polychromatic diffraction patterns of (*a*) a lysozyme crystal from the chip lys08 recorded at beamline ID09 with a JUNGFRAU 1M detector with an exposure time of 5 µs and (*b*) a proteinase K crystal from the chip protK04 with an exposure time of 1 µs. Magnified areas of the diffraction images indicated by a red square in images (*a*) and (*b*) are shown in (*c*) and (*e*). Images (*d*) and (*f*) show even higher magnifications of the areas indicated in (*c*) and (*e*) and highlight the achievable low background-scattering levels around the Bragg reflections at 3.1 Å in the case of lysozyme (*d*) and 3.6 Å in the case of proteinase K crystals (*f*).

All diffraction measurements were carried out at room temperature. Lysozyme diffraction data were collected at X-ray exposure times of 5 and 1 µs per crystal. Proteinase K data were collected only at an exposure time of 1 µs. With the beam parameters mentioned above and 3.5 × 10^9^ photons per 5 µs exposure and 7 × 10^8^ photons per 1 µs exposure, these exposure times correspond to X-ray doses of 500 and 100 Gy, respectively. At these doses, data should not be affected by radiation damage or sample heating effects, even without cryogenic cooling (Roedig *et al.*, 2016 ▸; Henderson, 1990 ▸). This was also confirmed experimentally by measuring multiple diffraction patterns at the same position of the chip and comparing the Bragg peaks at high resolution. Two example diffraction patterns of a lysozyme crystal and a proteinase K crystal are shown in Fig. 4 ▸. In total we collected diffraction data from ten chips. Scanning and data-collection parameters for every chip are provided in Table 1 ▸. The datasets, each from a separate chip, are labeled lys08 to lys15, and protK03 and protK04. On average, 36 000 diffraction patterns were collected per chip with a scanning time of about 150 s for an entire chip. This is longer than the 36 s of data-collection time because of the overhead of changing direction at the end of the scan. The hit fraction depends on the crystal-growth conditions. In the case of lysozyme crystals with 5 µs exposure, the average hit fraction was 30% and of these patterns 76% could be indexed, corresponding to an effective data-collection rate of 55 indexed patterns per second. For the lysozyme and proteinase K crystals measured with 1 µs exposure, the effective data-collection rate was lower with 28 and 9 indexed patterns per second, respectively, which is probably a result of a lower crystal density on the chip.

## 3. Data analysis   

Diffraction data were indexed and integrated using the *CrystFEL* software suite (White *et al.*, 2012 ▸), which was modified and extended to handle diffraction data recorded with 2.5% X-ray bandwidth and merged into different (sub-) datasets (see the Supporting information). A typical indexed diffraction pattern of a lysozyme crystal is displayed in Fig. S1 in the Supporting information, which shows a good correspondence between diffraction peaks and predicted Bragg spot positions. In Figs. S2 and S3 we further provide a comparison of the spot prediction and the resulting CC* values of the datasets obtained by using modified and un­modified versions of *CrystFEL*. An improvement in the data quality arising from the *CrystFEL* modifications is clearly visible. Structure refinements for all generated datasets were carried out using *PHENIX* (Adams *et al.*, 2010 ▸). PDB structures 6ftr and 5kxv served as starting models for lysozyme and proteinase K (Wiedorn *et al.*, 2018 ▸; Masuda *et al.*, 2017 ▸).

**Figure 5:**
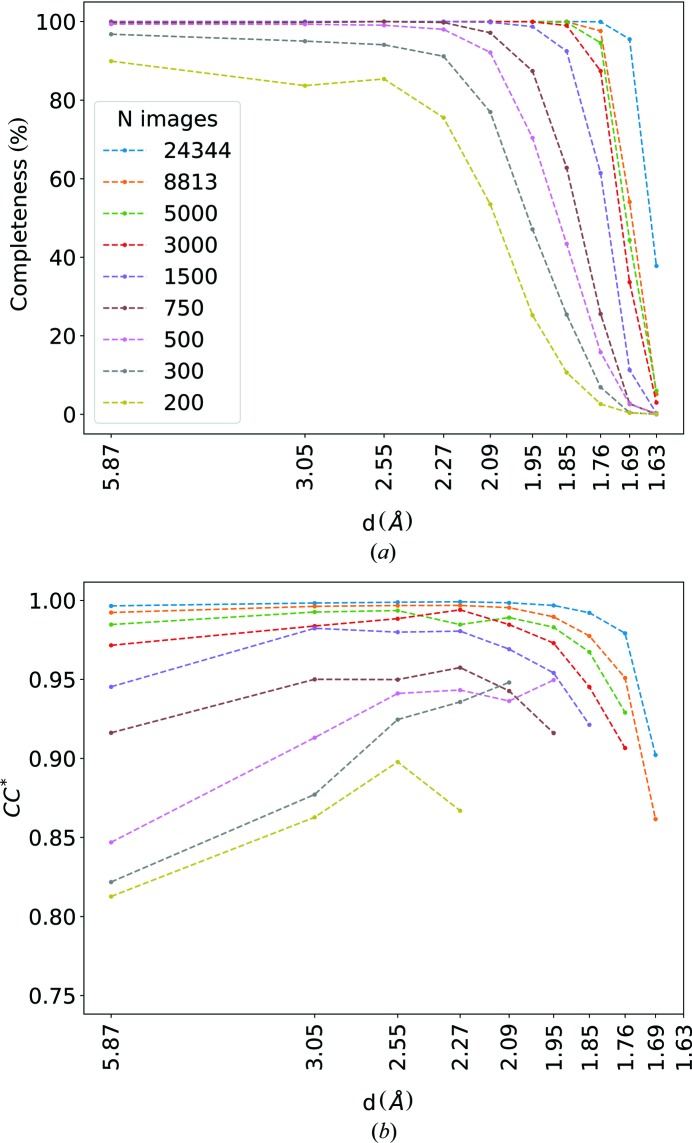
Completeness (*a*) and CC* (*b*) as a function of resolution for different numbers of merged patterns from the lysozyme datasets with 5 µs exposure time.

**Figure 6:**
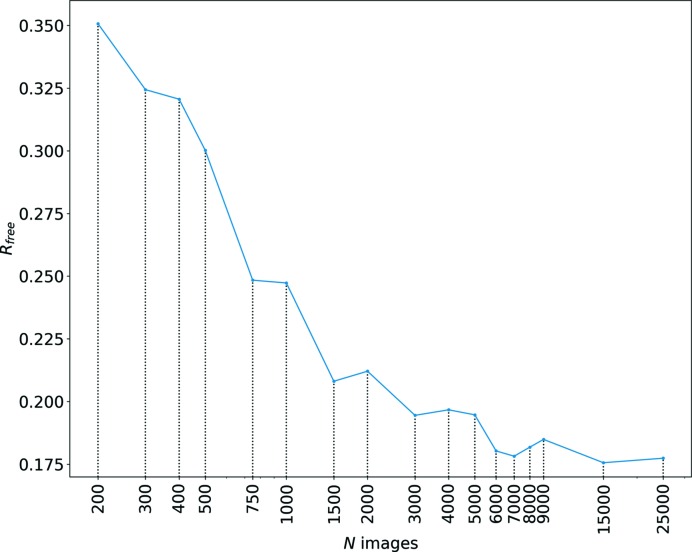
*R*_free_ as a function of the number of merged patterns from the lysozyme datasets with 5 µs exposure time.

The lysozyme diffraction datasets recorded with 5 µs exposure time (lys08, lys09, lys10) were further analyzed to determine the dependence of analysis metrics on the number of diffraction patterns collected. These three chips provided a total of 24 344 indexed diffraction patterns. We created eight subsets from this group, consisting of 200 to 15 000 randomly selected lysozyme diffraction patterns, plus the full dataset, which were all individually processed using *CrystFEL*. From these, structure refinements were then carried out with *PHENIX* (Adams *et al.*, 2010 ▸) which quantified data complete­ness and the correlation with the calculated structure factors, CC*, as a function of resolution, as shown in Figs. 5 ▸(*a*) and 5 ▸(*b*). Further metrics of some of the subsets are given in Table 2 ▸, and the free *R* factor, *R*_free_, is plotted in Fig. 6 ▸.

As seen in Figs. 5 ▸(*a*) and 5 ▸(*b*), all datasets containing more than 500 diffraction patterns show almost 100% completeness up to a resolution of 2.3 Å. Here, completeness is defined as the fraction of reflections in the resolution shell that have been integrated at least once regardless of their intensity. With increasing numbers of merged patterns, this metric extends to higher resolution. The completeness of the dataset containing all 24 344 frames remains close to 100% for resolutions of up to 1.7 Å. The dependence of CC* on the number of patterns exhibits a different behavior: only the datasets containing more than 2 500 patterns show CC* values larger than 0.95, which then falls off at resolutions higher than 2.3 Å. Again, the dataset containing all of the patterns shows the highest CC*, which also extends to the highest resolution. Interestingly, the datasets consisting of a smaller number of diffraction patterns show a low CC* not only for the high-resolution reflections but also for the low-resolution reflections.

As seen in Fig. 6 ▸, *R*_free_ first decreases rapidly with the increasing number of patterns, from 0.35 for 200 patterns to about 0.21 for 1 500 merged patterns. Beyond this number of patterns there is little improvement in this metric which decreases only slightly to 0.175 when all 24 000 patterns are included. Both the low CC* values for the low-resolution reflections and the relatively high *R*_free_ values for datasets consisting of less than 1 500 diffraction patterns can be better understood with reference to the Ewald construction of diffraction as illustrated in Fig. S4. The limiting spheres of the minimum and maximum wavelengths of the polychromatic radiation define a volume of reciprocal space where reflections occur. To the first order, this volume can be thought of as a wedge. At low resolution, the wedge is thinner than the peak width, giving mainly partial reflections that contribute to a variance in their measured intensities. This situation is similar to the case of monochromatic radiation. As the resolution increases, so too does the width of the wedge, which eventually is broader than the peak width. At this resolution and higher, reflections are predominantly fully recorded, giving measurements with less variance. These reflections therefore need measurements from fewer patterns to achieve a given confidence.

**Figure 7:**
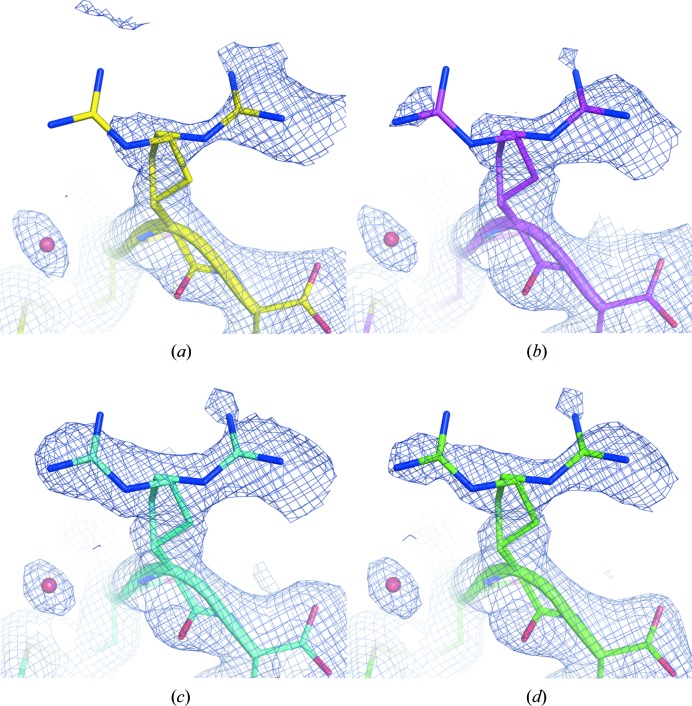
2*m* *F*_o_ − *D* *F*_c_ electron-density maps at 0.7σ level showing the poorly defined surface-residue Arg128 generated from datasets consisting of different numbers of merged diffraction patterns: (*a*) in yellow, 750 patterns; (*b*) in pink, 1 500 patterns; (*c*) in cyan, 3 000 patterns; and (*d*) in green, all patterns (24 344). Whereas in (*a*) and (*b*) the electron density is ambiguous, the merged datasets from 3 000 (*c*) and all 24 344 patterns (*d*) clearly reveal a second conformation of Arg128.

Example electron-density distributions around an Arg128 residue for some different subsets of the 5 µs lysozyme measurements are shown in Fig. 7 ▸. Whereas for the densities determined from 750 and 1 500 merged diffraction patterns only one conformation is visible [Figs. 7 ▸(*a*) and 7 ▸(*b*)], merges from 3 000 and all 24 344 patterns clearly reveal the occupation of a second conformation of residue 128. For other electron-density regions of the lysozyme structure we observe a similar trend of additional conformations appearing (see Figs. S5–S7). This is consistent with the observation of a relatively strong decrease of the *R*_free_ values for merges from 250 to 3 000 patterns and only a moderate further decrease when more diffraction patterns are considered.

**Figure 8:**
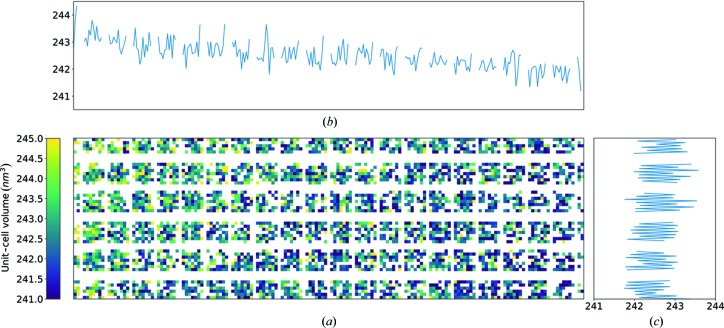
(*a*) Spatial distribution of the unit-cell volume of lysozyme crystals on the chip lys09 with dimensions of 33 × 12 mm, averaged in 2 × 2 bins. (*b*) Vertically averaged unit-cell volume as a function of the horizontal position on the chip. (*c*) Horizontally averaged unit-cell volume as a function of the vertical position on the chip.

In the method of fixed-target SX using a humidified gas stream to prevent the crystals from drying out, one consideration to be addressed is that the unit-cell parameters of the room-temperature crystals may vary, depending on their positions on the chip. For example, the unit-cell volumes of all the indexed lysozyme crystals from chip lys09 are found to be normally distributed, with a mean value of 242 400 Å^3^ and a standard deviation of 1 400 Å^3^ (see Fig. S8). This is about 1.5 times the standard deviation obtained from similarly prepared lysozyme crystals also measured at room temperature but in solution, in a liquid jet (Wiedorn *et al.*, 2018 ▸). In that case, measurements at the European XFEL yielded unit-cell volumes that were normally distributed with a mean of 237 100 Å^3^ and a standard deviation of 900 Å^3^. The spatial distribution of the unit-cell volume of crystals on the lys09 chip is plotted in Fig. 8 ▸. The unit-cell volume varies between 241 000 and 245 000 Å^3^, a relative change of about 1.6%, diminishing from the top-left corner to the bottom-right corner of the chip [see Figs. 8 ▸(*b*) and 8 ▸(*c*)]. This variation appears to be caused by an uneven humidity inside the chamber, which encloses the chip when it is measured. The chip is moved inside this humidity chamber when it is scanned. Figs. 3 ▸(*c*) and 3 ▸(*d*) show the start and end positions of the scan. Even though every crystal is measured in the same location within the chamber (the X-ray beam position), during the entire scan (and during alignment) the chip experiences a gradient of humidity because of the flow of humid air from one side of the chamber to the other.

Another effect that can be seen in Fig. 8 ▸(*c*) is an oscillation of the unit-cell volume in the *y* direction with a periodicity of two rows and a magnitude of 0.5% of the average unit-cell volume. This magnitude matches the overall gradient experienced in the longitudinal direction. Since the total horizontal line scan takes only about 0.3 s but the deceleration at the end of the line, vertical movement and acceleration at the beginning of the next line takes about 1 s, the crystals might have enough time to shrink while the chip stays in the right part of the chamber, where humidity is lower, and then partially recover when the chip is again in the left part, where humidity is higher. Another explanation for this variation could be a systematic shift of the stage in the *z* direction depending on the scan direction. However, we verified that this was not the case since the chip stays within the few micrometre depth-of-focus of the in-line microscope throughout the scan.

Structure refinements carried out with datasets containing merged intensities from different areas of the chip did not reveal any significant structural changes. Despite the systematic changes in unit-cell volume in a scan, the structures of lysozyme determined with the method of fixed-target SX with microsecond exposure times at a synchrotron are of similar quality to the recent structure determination carried out at the European XFEL (Wiedorn *et al.*, 2018 ▸; Grünbein *et al.*, 2018 ▸) using femtosecond exposure times. It should be noted here that with conventional crystallography of large lysozyme single crystals, much higher resolutions of up to 0.94 Å have been achieved (Sauter *et al.*, 2001 ▸).

## 4. Discussion   

Using the approach of high-speed fixed-target SX in combination with the new JUNGFRAU integrating pixel detector, we were able to collect a complete high-quality diffraction dataset at a frame rate of 1 kHz in less than 150 s. By using the method of on-chip crystallization (Lieske *et al.*, 2019 ▸), sample-preparation efforts were minimal as no additional crystal handling or manipulation steps such as pipetting are required. The crystals can be directly measured after removal of the mother liquor by blotting. The total time for preparation and measurement of one chip, including blotting, mounting of the chip on the goniometer, definition of the scan grid and data collection was about 10 minutes. After aligning the setup and establishing the data-collection procedure, we were able to measure ten chips in one hour, which directly translates into at least ten structure determinations.

In contrast to other sample-delivery methods, here the entire membrane area is systematically scanned through the X-ray beam guaranteeing that most of the material on the chip is exposed and contributes to the dataset. With about 10 000 crystals per chip with average dimensions of 50 µm this corresponds to a total amount of 1.6 mg of sample per chip, which is a fairly large amount of protein for a structural biology project. The main reason for using crystals of this size was to match the X-ray beam size of about 60 µm at ID09 at that time. The applied X-ray doses were as low as 100 Gy for the 1 µs exposure times, which is only about 16 times the LD_50_ dose for human beings of 6 Gy and more than five orders of magnitude less than typical doses of 50 MGy in cryocrystallography (Meents *et al.*, 2010 ▸; Owen *et al.*, 2006 ▸). This highlights the potential of the method for investigations of the undamaged structure of redox-sensitive metalloproteins at unprecedented low dose levels (Beitlich *et al.*, 2007 ▸; Yano *et al.*, 2005 ▸; Corbett *et al.*, 2007 ▸).

Using smaller polychromatic X-ray beams, soon available at several beamlines such as the MAX IV in Sweden, the ESRF in France and the Advanced Photon Source (APS) in the US, will allow a tremendous reduction in the amount of sample required for a pink-beam structure determination. Reducing the beam area from 60 × 60 µm used here to 10 × 10 µm while retaining the same number of photons per pulse will, on the one hand, increase the dose by a factor of 36 from 100 Gy to 3.6 kGy for a 1 µs exposure, but, on the other hand, should allow the collection of datasets of similar quality using only 1.6/36 mg = 44 µg of sample. X-ray doses of 20 kGy are still well below the room-temperature dose limit of about 300 kGy, and an amount of 44 µg corresponds to one single crystal with dimensions of 330 µm, a crystal size typical for structure determination with X-ray tubes in the laboratory (Roedig *et al.*, 2016 ▸).

As can be seen in Fig. 4 ▸, the background-scattering levels obtained in our measurements are very low, with the vast majority of the pixels having zero counts. This is a result of our low-background experimental setup (Meents *et al.*, 2017 ▸) in combination with the single-photon sensitivity of the JUNGFRAU detector. This leads to an improved signal-to-noise level of the data compared with that obtained with contemporary CCD detectors and thereby a higher overall data quality than usually achievable in conventional crystallographic experiments, especially for high-resolution reflections. Our fixed-target approach involves ‘naked’ crystals on the membrane, which are easily accessible for external manipulation. This makes the method ideally suited for ligand-binding studies and laser pump–probe experiments. Since any sealing of the chips with Mylar or Kapton foil is avoided, ligand solutions can be applied *in situ* to the crystals on the chip using, for example, micro-droplet generators. With crystals several micrometres in size, which will be measurable at beamlines with smaller beams and higher fluence to what we demonstrated here, diffusion times and hence the achievable time resolution in such experiments should be in the few millisecond range (Schmidt, 2013 ▸). Laser excitation of the crystals could be performed using illumination through the in-line sample-viewing microscope. Here again, the absence of any sealing material avoids unwanted reflections and scattering of the pump laser light that might otherwise cause unwanted pre-exposure of the neighboring crystals.

In comparison with diffraction experiments with polychromatic X-rays using the full 5% bandwidth of an undulator harmonic (Meents *et al.*, 2017 ▸), the spectrum produced by the multilayer monochromator is approximately symmetric, which greatly facilitates data processing. The indexing and data-integration procedures developed and established here will be integrated into an upcoming version of the *CrystFEL* data-processing suite, and will be published elsewhere.

With an achievable time resolution in the microsecond range and even down to below a nanosecond with single bunches (Meents *et al.*, 2017 ▸), the opportunities for time-resolved experiments at synchrotrons should be further developed. Many SX experiments currently performed at XFEL sources tend to use crystals that are large enough to give measurable diffraction signals at high-intensity synchrotron beamlines. Our method represents an attractive alternative for such experiments.

The method of pink-beam SX at synchrotron sources allows the collection of ultra-low-dose datasets of proteins from small crystals. This should enable almost damage-free structures of redox-sensitive proteins. It avoids cryopreparation and allows the collection of data at physiologically relevant temperatures for the study of conformational flexibility (Fraser *et al.*, 2011 ▸) and time-resolved measurements of structures undergoing triggered reactions. With data-collection times of less than a minute for a complete dataset, it is further well suited for time-efficient and systematic screening of pharmaceutical compounds. In the future, we will perform time-resolved measurements by this method to study irreversible processes such as enzyme reactions. Automatic exchange of chips with a robotic arm in combination with fully automatic identification of the scanning grid should further increase the throughput of our approach.

## 5. Related literature   

The following references are cited in the Supporting information for this article: Afonine *et al.* (2012 ▸); Cammarata *et al.* (2009 ▸); Chen *et al.* (2010 ▸); Emsley *et al.* (2010 ▸); Göries *et al.* (2016 ▸); McCoy *et al.* (2007 ▸); Redford *et al.* (2018 ▸); Urzhumtseva (2009 ▸); White *et al.* (2016 ▸); Yefanov *et al.* (2015 ▸).

## Supplementary Material

Supporting information. DOI: 10.1107/S205225251900914X/ec5014sup1.pdf

PDB reference: lysozyme, 5 µs exposure, 750 patterns merged, 6qy2

PDB reference: lysozyme, 5 µs exposure, 1 500 patterns merged, 6qy1

PDB reference: lysozyme, 5 µs exposure, 24 344 patterns merged (3 chips), 6qxw

PDB reference: Proteinase K, 1 µs exposure, 1 585 patterns merged (2 chips), 6qxv

PDB reference: lysozyme, 1 µs exposure, 14 793 patterns merged, 6qy4

PDB reference: lysozyme, 5 µs exposure, 3 000 patterns merged, 6qy0

## Figures and Tables

**Table 1:** Chip-scanning parameters for 1 kHz fixed-target data collection with the Roadrunner II goniometer All chips were scanned with a horizontal scanning speed of 100 mm s^−1^.

Chip name	lys08	lys09	lys10	lys11	lys12	lys13	lys14	lys15	protK3	protK4
Exposure time (µs)	5	5	5	1	1	1	1	1	1	1
No. of horizontal scan points	331†	331†	331†	333†	331†	151‡	163‡	156‡	310†	156‡
No. of vertical scan points	116	105	104	116	105	24‡	116	105	114	105
Total no. of scan points	38396	34752	34423	38628	34754	3580	18907	16365	35340	16379
No. of hits	12209	12512	7489	5376	4621	937	4707	3443	2538	640
No. of indexed and merged hits	9238	8813	6293	4448	3885	762	3386	2312	1366	219
Total scanning time (s)	158	143	142	158	143	28	139	125	153	125
Hits per second	77	87	53	34	32	33	34	28	17	5.1
Indexed patterns per second	58	62	44	28	27	27	24	18	8.9	1.8
Effective scanning rate (frames s^−1^)	243	243	243	244	243	130	137	131	231	132

† 15 scan points at the beginning and end of every line were used for acceleration and deceleration of the linear axis, so the total horizontal scanning range slightly exceeds the chip lengths.

‡ These chips were only partially scanned.

**Table 2:** Data-collection and structure-refinement parameters for the six resulting polychromatic lysozyme and proteinase K datasets Values in parentheses are for the outer shell.

	Lysozyme, all patterns, 5 µs exposure	Lysozyme, 3000 patterns, 5 µs exposure	Lysozyme, 1500 patterns, 5 µs exposure	Lysozyme, 750 patterns, 5 µs exposure	Lysozyme, all patterns, 1 µs exposure	Proteinase K, two chips, 1 µs exposure
Space group	*P*4_3_2_1_2					*P*4_3_2_1_2
Unit-cell parameters *a*, *b*, *c* (Å) α, β, γ (°)	79.8 (0.2), 79.9 (0.2), 38.0 (0.1), 90.0 (0.1), 90.0 (0.1), 90.0 (0.1)					68.6 (0.2), 68.6 (0.2), 104.5 (0.5), 90 (0.2), 90 (0.1), 90 (0.2)
Exposure time (µs)	5	5	5	5	1	1
No. of merged images	24344	3000	1500	750	14793	1585
Multiplicity	315.5	39.7	20.9	11.4	162.8	23.1
〈*I*/σ(*I*)〉	13.66	6.06	4.20	3.84	9.12	4.60
CC*	0.9973	0.9823	0.9668	0.9427	0.9973	0.9647
*R*_split_ (%)	6.01	18.1	24.8	34.8	7.55	24.19
Wilson *B* factor (Å^2^)	19.48	19.50	19.92	19.56	20.15	22.79
Resolution range (Å)	19.37–1.7 (1.761–1.7)	19.37–1.7 (1.761–1.7)	19.37–1.7 (1.761–1.7)	19.37–1.7 (1.761–1.7)	19.37–1.7 (1.761–1.7)	21.7–1.94 (2.009–1.94)
Unique reflections	14032 (1354)	13698 (1064)	13042 (642)	11637 (221)	11142 (1088)	18492 (1402)
Completeness (%)	99.89 (99.85)	96.53 (69.42)	91.36 (37.55)	81.27 (11.66)	95.21 (58.03)	96.25 (73.54)
Reflections used in refinement	14032 (1354)	13676 (1048)	12997 (622)	11569 (204)	11140 (1086)	18430 (1381)
Reflections used for *R*_free_	1382 (133)	1353 (108)	1290 (62)	1138 (17)	1097 (107)	1767 (133)
*R*_work_	0.1486 (0.1943)	0.1654 (0.2826)	0.1863 (0.3089)	0.2152 (0.3066)	0.1560 (0.2028)	0.1721 (0.2345)
*R*_free_	0.1742 (0.2193)	0.1944 (0.3302)	0.2062 (0.3405)	0.2417 (0.3074)	0.1830 (0.2429)	0.2293 (0.2844)
No. of non-hydrogen atoms	1166	1166	1166	1166	1139	2493
Macromolecules	1077	1077	1077	1077	1050	2246
Ligands	8	8	8	8	8	60
Protein residues	129	129	129	129	129	279
RMS (bonds)	0.010	0.009	0.004	0.003	0.007	0.010
RMS (angles)	1.05	0.99	0.69	0.55	0.81	1.31
Ramachandran plot						
Favored (%)	99	99	99	99	99	97.47
Allowed (%)	0.72	0.72	1.4	1.4	0.74	2.53
Outliers (%)	0	0	0	0	0	0.00
Rotamer outliers (%)	0	0	0	0	0	2.09
Clashscore	2.32	2.78	1.39	1.39	1.91	4.49
Average *B* factor (Å^2^)	23.28	22.97	23.15	23.98	22.36	24.68
Macromolecules	22.28	22.06	22.24	23.25	21.57	22.50
Ligands	32.55	31.54	30.98	29.21	28.77	66.62
Solvent	35.75	34.27	34.45	33.22	32.05	37.35

## References

[bb1] Adams, P. D., Afonine, P. V., Bunkóczi, G., Chen, V. B., Davis, I. W., Echols, N., Headd, J. J., Hung, L.-W., Kapral, G. J., Grosse-Kunstleve, R. W., McCoy, A. J., Moriarty, N. W., Oeffner, R., Read, R. J., Richardson, D. C., Richardson, J. S., Terwilliger, T. C. & Zwart, P. H. (2010). *Acta Cryst.* D**66**, 213–221. 10.1107/S0907444909052925 PMC2815670 20124702

[bb39] Afonine, P. V., Grosse-Kunstleve, R. W., Echols, N., Headd, J. J., Moriarty, N. W., Mustyakimov, M., Terwilliger, T. C., Urzhumtsev, A., Zwart, P. H. & Adams, P. D. (2012). *Acta Cryst.* D**68**, 352–367. 10.1107/S0907444912001308 PMC3322595 22505256

[bb2] Allahgholi, A., Becker, J., Bianco, L., Delfs, A., Dinapoli, R., Ariño-Estrada, G., Goettlicher, P., Graafsma, H., Greiffenberg, D., Hirsemann, H., Jack, S., Klanner, R., Klyuev, A., Krueger, H., Lange, S., Marras, A., Mezza, D., Mozzanica, A., Poehlsen, J., Rah, S., Xia, Q., Schmitt, B., Schwandt, J., Sheviakov, I., Shi, X., Smoljanin, S., Trunk, U., Zhang, J. & Zimmer, M. (2016). *J. Instrum.* **11**, C01057.

[bb28] Beitlich, T., Kühnel, K., Schulze-Briese, C., Shoeman, R. L. & Schlichting, I. (2007). *J. Synchrotron Rad.* **14**, 11–23. 10.1107/S0909049506049806 17211068

[bb3] Beyerlein, K. R., Dierksmeyer, D., Mariani, V., Kuhn, M., Sarrou, I., Ottaviano, A., Awel, S., Knoska, J., Fuglerud, S., Jönsson, O., Stern, S., Wiedorn, M. O., Yefanov, O., Adriano, L., Bean, R., Burkhardt, A., Fischer, P., Heymann, M., Horke, D. A., Jungnickel, K. E. J., Kovaleva, E., Lorbeer, O., Metz, M., Meyer, J., Morgan, A., Pande, K., Panneerselvam, S., Seuring, C., Tolstikova, A., Lieske, J., Aplin, S., Roessle, M., White, T. A., Chapman, H. N., Meents, A. & Oberthuer, D. (2017). *IUCrJ*, **4**, 769–777. 10.1107/S2052252517013124 PMC5668862 29123679

[bb4] Broennimann, Ch., Eikenberry, E. F., Henrich, B., Horisberger, R., Huelsen, G., Pohl, E., Schmitt, B., Schulze-Briese, C., Suzuki, M., Tomizaki, T., Toyokawa, H. & Wagner, A. (2006). *J. Synchrotron Rad.* **13**, 120–130. 10.1107/S0909049505038665 16495612

[bb34] Cammarata, M., Eybert, L., Ewald, F., Reichenbach, W., Wulff, M., Anfinrud, P., Schotte, F., Plech, A., Kong, Q., Lorenc, M., Lindenau, B., Räbiger, J. & Polachowski, S. (2009). *Rev. Sci. Instrum.* **80**, 015101. 10.1063/1.3036983 19191457

[bb5] Chapman, H. N., Fromme, P., Barty, A., White, T. A., Kirian, R. A., Aquila, A., Hunter, M. S., Schulz, J., DePonte, D. P., Weierstall, U., Doak, R. B., Maia, F. R. N. C., Martin, A. V., Schlichting, I., Lomb, L., Coppola, N., Shoeman, R. L., Epp, S. W., Hartmann, R., Rolles, D., Rudenko, A., Foucar, L., Kimmel, N., Weidenspointner, G., Holl, P., Liang, M., Barthelmess, M., Caleman, C., Boutet, S., Bogan, M. J., Krzywinski, J., Bostedt, C., Bajt, S., Gumprecht, L., Rudek, B., Erk, B., Schmidt, C., Hömke, A., Reich, C., Pietschner, D., Strüder, L., Hauser, G., Gorke, H., Ullrich, J., Herrmann, S., Schaller, G., Schopper, F., Soltau, H., Kühnel, K. U., Messerschmidt, M., Bozek, J. D., Hau-Riege, S. P., Frank, M., Hampton, C. Y., Sierra, R. G., Starodub, D., Williams, G. J., Hajdu, J., Timneanu, N., Seibert, M. M., Andreasson, J., Rocker, A., Jönsson, O., Svenda, M., Stern, S., Nass, K., Andritschke, R., Schröter, C. D., Krasniqi, F., Bott, M., Schmidt, K. E., Wang, X., Grotjohann, I., Holton, J. M., Barends, T. R. M., Neutze, R., Marchesini, S., Fromme, R., Schorb, S., Rupp, D., Adolph, M., Gorkhover, T., Andersson, I., Hirsemann, H., Potdevin, G., Graafsma, H., Nilsson, B. & Spence, J. C. H. (2011). *Nature*, **470**, 73–77.

[bb35] Chen, V. B., Arendall, W. B., Headd, J. J., Keedy, D. A., Immormino, R. M., Kapral, G. J., Murray, L. W., Richardson, J. S. & Richardson, D. C. (2010). *Acta Cryst.* D**66**, 12–21. 10.1107/S0907444909042073 PMC2803126 20057044

[bb6] Corbett, M. C., Latimer, M. J., Poulos, T. L., Sevrioukova, I. F., Hodgson, K. O. & Hedman, B. (2007). *Acta Cryst.* D**63**, 951–960. 10.1107/S0907444907035160 17704563

[bb36] Emsley, P., Lohkamp, B., Scott, W. G. & Cowtan, K. (2010). *Acta Cryst.* D**66**, 486–501. 10.1107/S0907444910007493 PMC2852313 20383002

[bb7] Fraser, J. S., van den Bedem, H., Samelson, A. J., Lang, P. T., Holton, J. M., Echols, N. & Alber, T. (2011). *Proc. Natl Acad. Sci. USA*, **108**, 16247–16252. 10.1073/pnas.1111325108 PMC3182744 21918110

[bb37] Göries, D., Dicke, B., Roedig, P., Stübe, N., Meyer, J., Galler, A., Gawelda, W., Britz, A., Geßler, P., Sotoudi Namin, H., Beckmann, A., Schlie, M., Warmer, M., Naumova, M., Bressler, C., Rübhausen, M., Weckert, E. & Meents, A. (2016). *Rev. Sci. Instrum.* **87**, 053116. 10.1063/1.4948596 27250401

[bb8] Grünbein, M. L., Bielecki, J., Gorel, A., Stricker, M., Bean, R., Cammarata, M., Dörner, K., Fröhlich, L., Hartmann, E., Hauf, S., Hilpert, M., Kim, Y., Kloos, M., Letrun, R., Messerschmidt, M., Mills, G., Nass Kovacs, G., Ramilli, M., Roome, C. M., Sato, T., Scholz, M., Sliwa, M., Sztuk-Dambietz, J., Weik, M., Weinhausen, B., Al-Qudami, N., Boukhelef, D., Brockhauser, S., Ehsan, W., Emons, M., Esenov, S., Fangohr, H., Kaukher, A., Kluyver, T., Lederer, M., Maia, L., Manetti, M., Michelat, T., Münnich, A., Pallas, F., Palmer, G., Previtali, G., Raab, N., Silenzi, A., Szuba, J., Venkatesan, S., Wrona, K., Zhu, J., Doak, R. B., Shoeman, R. L., Foucar, L., Colletier, J. P., Mancuso, A. P., Barends, T. R. M., Stan, C. A. & Schlichting, I. (2018). *Nat. Commun.* **9**, 3487.

[bb9] Grünbein, M. L. & Nass Kovacs, G. (2019). *Acta Cryst.* D**75**, 178–191. 10.1107/S205979831801567X PMC6400261 30821706

[bb10] Hart, P., Boutet, S., Carini, G., Dubrovin, M., Duda, B., Fritz, D., Haller, G., Herbst, R., Herrmann, S., Kenney, C., Kurita, N., Lemke, H., Messerschmidt, M., Nordby, M., Pines, J., Schafer, D., Swift, M., Weaver, M., Williams, G., Zhu, D. L., Van Bakel, N. & Morse, J. (2012). *Proc. SPIE*, **8504**, 85040C.

[bb11] Henderson, R. (1990). *Proc. R. Soc. B Biol. Sci.* **241**, 6–8.

[bb12] Hunter, M. S., Segelke, B., Messerschmidt, M., Williams, G. J., Zatsepin, N. A., Barty, A., Benner, W. H., Carlson, D. B., Coleman, M., Graf, A., Hau-Riege, S. P., Pardini, T., Seibert, M. M., Evans, J., Boutet, S. & Frank, M. (2014). *Sci. Rep.* **4**, 6026. 10.1038/srep06026 PMC4129423 25113598

[bb13] Leonarski, F., Redford, S., Mozzanica, A., Lopez-Cuenca, C., Panepucci, E., Nass, K., Ozerov, D., Vera, L., Olieric, V., Buntschu, D., Schneider, R., Tinti, G., Froejdh, E., Diederichs, K., Bunk, O., Schmitt, B. & Wang, M. (2018). *Nat. Methods*, **15**, 799–804. 10.1038/s41592-018-0143-7 30275593

[bb14] Lieske, J., Cerv, M., Kreida, S., Komadina, D., Fischer, J., Barthelmess, M., Fischer, P., Pakendorf, T., Yefanov, O., Mariani, V., Seine, T., Ross, B. H., Crosas, E., Lorbeer, O., Burkhardt, A., Lane, T. J., Guenter, S., Bergholdt, J., Schoen, S., Törnroth-Horsefield, S., Chapman, H. N. & Meents, A. (2019). *IUCrJ*, **6**, 714–728. 10.1107/S2052252519007395 PMC6608620 31316815

[bb15] Martiel, I., Müller-Werkmeister, H. M. & Cohen, A. E. (2019). *Acta Cryst.* D**75**, 160–177. 10.1107/S2059798318017953 PMC6400256 30821705

[bb16] Martin-Garcia, J. M., Zhu, L., Mendez, D., Lee, M.-Y., Chun, E., Li, C., Hu, H., Subramanian, G., Kissick, D., Ogata, C., Henning, R., Ishchenko, A., Dobson, Z., Zhang, S., Weierstall, U., Spence, J. C. H., Fromme, P., Zatsepin, N. A., Fischetti, R. F., Cherezov, V. & Liu, W. (2019). *IUCrJ*, **6**, 412–425. 10.1107/S205225251900263X PMC6503920 31098022

[bb17] Masuda, T., Suzuki, M., Inoue, S., Song, C., Nakane, T., Nango, E., Tanaka, R., Tono, K., Joti, Y., Kameshima, T., Hatsui, T., Yabashi, M., Mikami, B., Nureki, O., Numata, K., Iwata, S. & Sugahara, M. (2017). *Sci. Rep.* **7**, 45604. 10.1038/srep45604 PMC5374539 28361898

[bb38] McCoy, A. J., Grosse-Kunstleve, R. W., Adams, P. D., Winn, M. D., Storoni, L. C. & Read, R. J. (2007). *J. Appl. Cryst.* **40**, 658–674. 10.1107/S0021889807021206 PMC2483472 19461840

[bb18] Meents, A., Gutmann, S., Wagner, A. & Schulze-Briese, C. (2010). *Proc. Natl Acad. Sci.* **107**, 1094–1099. 10.1073/pnas.0905481107 PMC2798883 20080548

[bb19] Meents, A., Wiedorn, M. O., Srajer, V., Henning, R., Sarrou, I., Bergtholdt, J., Barthelmess, M., Reinke, P. Y. A., Dierksmeyer, D., Tolstikova, A., Schaible, S., Messerschmidt, M., Ogata, C. M., Kissick, D. J., Taft, M. H., Manstein, D. J., Lieske, J., Oberthuer, D., Fischetti, R. F. & Chapman, H. N. (2017). *Nat. Commun.* **8**, 1281. 10.1038/s41467-017-01417-3 PMC5668288 29097720

[bb20] Mozzanica, A., Bergamaschi, A., Brueckner, M., Cartier, S., Dinapoli, R., Greiffenberg, D., Jungmann-Smith, J., Maliakal, D., Mezza, D., Ramilli, M., Ruder, C., Schaedler, L., Schmitt, B., Shi, X. & Tinti, G. (2016). *J. Instrum.* **11**, C02047.

[bb21] Owen, R. L., Rudiño-Piñera, E. & Garman, E. F. (2006). *Proc. Natl Acad. Sci.* **103**, 4912–4917. 10.1073/pnas.0600973103 PMC1458769 16549763

[bb40] Redford, S., Andrä, M., Barten, R., Bergamaschi, A., Brückner, M., Dinapoli, R., Fröjdh, E., Greiffenberg, D., Lopez-Cuenca, C., Mezza, D., Mozzanica, A., Ramilli, M., Ruat, M., Ruder, C., Schmitt, B., Shi, X., Thattil, D., Tinti, G., Vetter, S. & Zhang, J. (2018). *J. Instrum.*, **13**, C01027.

[bb22] Roedig, P., Duman, R., Sanchez-Weatherby, J., Vartiainen, I., Burkhardt, A., Warmer, M., David, C., Wagner, A. & Meents, A. (2016). *J. Appl. Cryst.* **49**, 968–975. 10.1107/S1600576716006348 PMC4886986 27275143

[bb23] Roedig, P., Ginn, H. M., Pakendorf, T., Sutton, G., Harlos, K., Walter, T. S., Meyer, J., Fischer, P., Duman, R., Vartiainen, I., Reime, B., Warmer, M., Brewster, A. S., Young, I. D., Michels-Clark, T., Sauter, N. K., Kotecha, A., Kelly, J., Rowlands, D. J., Sikorsky, M., Nelson, S., Damiani, D. S., Alonso-Mori, R., Ren, J., Fry, E. E., David, C., Stuart, D. I., Wagner, A. & Meents, A. (2017). *Nat. Methods*, **14**, 805–810. 10.1038/nmeth.4335 PMC5588887 28628129

[bb24] Roedig, P., Vartiainen, I., Duman, R., Panneerselvam, S., Stübe, N., Lorbeer, O., Warmer, M., Sutton, G., Stuart, D. I., Weckert, E., David, C., Wagner, A. & Meents, A. (2015). *Sci. Rep.* **5**, 10451. 10.1038/srep10451 PMC4448500 26022615

[bb25] Sauter, C., Otálora, F., Gavira, J.-A., Vidal, O., Giegé, R. & García-Ruiz, J. M. (2001). *Acta Cryst.* D**57**, 1119–1126. 10.1107/s0907444901008873 11468395

[bb26] Schlichting, I. (2015). *IUCrJ*, **2**, 246–255. 10.1107/S205225251402702X PMC4392417 25866661

[bb27] Schmidt, M. (2013). *Adv. Condens. Matter Phys.* **2013**, 1–10.

[bb29] Stellato, F., Oberthür, D., Liang, M., Bean, R., Gati, C., Yefanov, O., Barty, A., Burkhardt, A., Fischer, P., Galli, L., Kirian, R. A., Meyer, J., Panneerselvam, S., Yoon, C. H., Chervinskii, F., Speller, E., White, T. A., Betzel, C., Meents, A. & Chapman, H. N. (2014). *IUCrJ*, **1**, 204–212. 10.1107/S2052252514010070 PMC4107920 25075341

[bb41] Urzhumtseva, L., Afonine, P. V., Adams, P. D. & Urzhumtsev, A. (2009). *Acta Cryst.* D**65**, 297–300. 10.1107/S0907444908044296 PMC2651759 19237753

[bb30] White, T. A., Barty, A., Stellato, F., Holton, J. M., Kirian, R. A., Zatsepin, N. A. & Chapman, H. N. (2013). *Acta Cryst.* D**69**, 1231–1240. 10.1107/S0907444913013620 PMC3689526 23793149

[bb31] White, T. A., Kirian, R. A., Martin, A. V., Aquila, A., Nass, K., Barty, A. & Chapman, H. N. (2012). *J. Appl. Cryst.* **45**, 335–341.

[bb42] White, T. A., Mariani, V., Brehm, W., Yefanov, O., Barty, A., Beyerlein, K. R., Chervinskii, F., Galli, L., Gati, C., Nakane, T., Tolstikova, A., Yamashita, K., Yoon, C. H., Diederichs, K. & Chapman, H. N. (2016). *J. Appl. Cryst.* **49**, 680–689. 10.1107/S1600576716004751 PMC4815879 27047311

[bb32] Wiedorn, M. O., Oberthür, D., Bean, R., Schubert, R., Werner, N., Abbey, B., Aepfelbacher, M., Adriano, L., Allahgholi, A., Al-Qudami, N., Andreasson, J., Aplin, S., Awel, S., Ayyer, K., Bajt, S., Barák, I., Bari, S., Bielecki, J., Botha, S., Boukhelef, D., Brehm, W., Brockhauser, S., Cheviakov, I., Coleman, M. A., Cruz-Mazo, F., Danilevski, C., Darmanin, C., Doak, R. B., Domaracky, M., Dörner, K., Du, Y., Fangohr, H., Fleckenstein, H., Frank, M., Fromme, P., Gañán-Calvo, A. M., Gevorkov, Y., Giewekemeyer, K., Ginn, H. M., Graafsma, H., Graceffa, R., Greiffenberg, D., Gumprecht, L., Göttlicher, P., Hajdu, J., Hauf, S., Heymann, M., Holmes, S., Horke, D. A., Hunter, M. S., Imlau, S., Kaukher, A., Kim, Y., Klyuev, A., Knoška, J., Kobe, B., Kuhn, M., Kupitz, C., Küpper, J., Lahey-Rudolph, J. M., Laurus, T., Le Cong, K., Letrun, R., Xavier, P. L., Maia, L., Maia, F. R. N. C., Mariani, V., Messerschmidt, M., Metz, M., Mezza, D., Michelat, T., Mills, G., Monteiro, D. C. F., Morgan, A., Mühlig, K., Munke, A., Münnich, A., Nette, J., Nugent, K. A., Nuguid, T., Orville, A. M., Pandey, S., Pena, G., Villanueva-Perez, P., Poehlsen, J., Previtali, G., Redecke, L., Riekehr, W. M., Rohde, H., Round, A., Safenreiter, T., Sarrou, I., Sato, T., Schmidt, M., Schmitt, B., Schönherr, R., Schulz, J., Sellberg, J. A., Seibert, M. M., Seuring, C., Shelby, M. L., Shoeman, R. L., Sikorski, M., Silenzi, A., Stan, C. A., Shi, X., Stern, S., Sztuk-Dambietz, J., Szuba, J., Tolstikova, A., Trebbin, M., Trunk, U., Vagovic, P., Ve, T., Weinhausen, B., White, T. A., Wrona, K., Xu, C., Yefanov, O., Zatsepin, N., Zhang, J., Perbandt, M., Mancuso, A. P., Betzel, C., Chapman, H. & Barty, A. (2018). *Nat. Commun.* **9**, 4025.

[bb33] Yano, J., Sauer, K., Pushkar, Y., Latimer, M. J., Zouni, A., Messinger, J., Yachandra, V. K., Biesiadka, J., Irrgang, K.-D., Kern, J., Glatzel, P., Loll, B. & Bergmann, U. (2005). *Proc. Natl Acad. Sci.* **102**, 12047–12052. 10.1073/pnas.0505207102 PMC1186027 16103362

[bb43] Yefanov, O., Mariani, V., Gati, C., White, T. A., Chapman, H. N. & Barty, A. (2015). *Opt. Express.* **23**, 28459. 10.1364/OE.23.028459 PMC4646514 26561117

