# Supplementary material for: 1 kHz fixed-target serial crystallography using a multilayer monochromator and an integrating pixel detector
Source: IUCrJ. 2019 Aug 17;6(Pt 5):927–37. doi: 10.1107/S205225251900914X (PMC6760437; doi:10.1107/S205225251900914X)
Supplement: Supplementary file 1 [file m-06-00927-sup1.pdf]

IUCrJ

Volume 6 (2019)

Supporting information for article:

1 kHz fixed-target serial crystallography using a multilayer monochromator and an integrating pixel detector

A. Tolstikova, M. Levantino, O. Yefanov, V. Hennicke, P. Fischer, J. Meyer, A. Mozzanica, S. Redford, E. Crosas, N. L. Opara, M. Barthelmess, J. Lieske, D. Oberthuer, E. Wator, I. Mohacsi, M. Wulff, B. Schmitt, H. N. Chapman and A. Meents

S1. Crystal growth

Hen egg white lysozyme and *Tritirachium album* proteinase K, purchased from Sigma-Aldrich and Carl Roth, respectively, were grown directly on Roadrunner II chips by vapour-diffusion using the on-chip crystallization method (Lieske *et al.*, 2019). A solution of 40-60mg/ml lysozyme dissolved in 50mM sodium acetate pH 3.5 was mixed in a 1:1 ratio with precipitant solution (50mM sodium acetate 3.5, 0.75M sodium chloride, 30% ethylene glycol, 11.25% polyethylene glycol 400). For each chip, a volume of 100µl of this mixture was applied and equilibrated against a reservoir of 7ml precipitant solution. Lysozyme crystals appeared at 4°C overnight. Proteinase K was crystallized accordingly by mixing 20mg/ml protein in 0.1M CHC buffer pH 7.0 and 10mM calcium chloride 1:1 with precipitant solution containing 0.1M CHC buffer pH 6.6, 0.7M ammonium sulphate and 10mM calcium chloride. Proteinase K crystals were grown by equilibrating 100µl protein-precipitant mixture on a Roadrunner II chip against a reservoir of 7ml precipitant solution overnight at room temperature.

S2. Mounting and handling of chips

All sample manipulation steps of Roadrunner II chips were performed in a bespoke sample handling chamber, which provides a well-defined high-humidity environment to prevent the crystals from dehydrating. For on-chip crystallization of the sample, holders were mounted in the humidity chamber and the growth solution was removed by blotting with a filter paper from the lower side of the chip as described in reference 18. After blotting, the chips were protected with a cover and transferred to the beamline where they were inserted into the Roadrunner II measurement chamber whilst the protective cover was retracted from the chip area.

S3. Data collection at ID09

X-ray diffraction data were collected at the ID09 beamline of European Synchrotron Radiation Facility (ESRF) while the storage ring was operated in uniform filling mode. The Roadrunner II diffractometer was installed in the sample area and synchronized with the arrival of X-ray pulses and the JUNGFRAU 1M detector readout (1 KHz repetition rate) by means of an FPGA synchronization board (see further details below).

S4. Beamline details

The U17 in-vacuum undulator of the ID09 beamline was set to a 6 mm gap, which resulted in an X-ray spectrum with an asymmetric peak at 15.2 keV and a high-energy broad component at ~27 keV. By means of a Ruthenium multilayer monochromator the central part of the main X-ray peak was selected, providing a more symmetric spectrum centered at 15.1 keV with a relative bandwidth of 2.5% and no high-energy component. The X-ray beam was focused at the sample position with a Platinum-coated toroidal mirror at an incidence angle of 2.608 mrad and slitted down so as to obtain a 0.06 mm (FWHM) round spot at the sample position.

In order to exploit the high repetition rate (~1 kHz) achievable by the Roadrunner II diffractometer and the JUNGFRAU 1M detector, it was necessary to select X-ray pulses at the same repetition rate and to vary their duration to vary the X-ray dose to the crystals. This was achieved by the use of two choppers and a fast shutter. The first chopper (heat load chopper, developed at the ESRF) was located upstream of the ID09 beamline and selected ~80 µs duration X-ray pulses at 1 kHz. The second chopper (high speed chopper, Forschungszentrum Jülich) was operated in the so-called "tunnel-less" mode (Cammarata *et al.*, 2009) and used to select X-ray pulses with variable duration in the 1-25 µs range. The chopper slit width gradually increases with its horizontal position, so a horizontal translation of the whole device allows adjusting the exposure time. Finally, a fast shutter was used to transmit X-rays in synchrony with the horizontal scan of the chip, so that X-rays were blanked to avoid exposing regions on the chip outside the desired scan region. The synchronization signals for the two choppers, the fast shutter and the Roadrunner II motion, together with the gate pulse for the JUNGFRAU 1M detector were produced by a N354 FPGA board developed at the ESRF, which is capable of producing bursts of pulses at the 360th sub-harmonic of the synchrotron orbit frequency (~355 kHz).

S5. Roadrunner goniometer

A technical drawing of the Roadrunner II goniometer is shown in Figure 3a. It consists of four main units: An inline sample viewing microscope based on a Schwarzsschild microscope objective with a drill hole for the X-rays to pass through, similar to the device described in (Göries *et al.*, 2016). The goniometer unit consists of a high-speed linear axis for fast sample translation with speeds of up to 120 mm/s which is mounted on a *y*,*z* centering stage, which allows the sample to be positioned in center of the rotation axis. This whole scanning unit is placed on a φ-rotation stage, which can be used to rotate the chips with respect to the incident beam direction and thereby avoiding effects due to preferred orientation of the crystals on the chip.

S6. Humidity setup

The measurement chamber of the Roadunner goniometer (shown in Figure 3c and 3d) was constantly flushed with 10 l/min of humidified helium gas with a relative humidity > 95% in order to prevent the crystals from drying out. Humidification of the helium gas was achieved by guiding it through four water filled glass bottles using bubbling stones to increase the efficiency of the humidification process. A fraction of 10% of the helium gas stream was guided to a mirror hygrometer ('OptiDew') to permanently monitor the humidity.

S7. JUNGFRAU detector

Diffraction patterns were recorded with a JUNGFRAU 1M detector which consists of two 1024x512 pixel modules with a pixel pitch of 75um and a effective area per module of 77.25x38.5mm2. The detector provides single photon sensitivity in combination with a dynamic range of about 8000 photons at the 15keV energy used in our experiment. The high dynamic range is possible thanks to the automatic gain capability of each individual pixel: each pixel adjusts (lowers) its gain to cope to the incoming instantaneous signal (Mozzanica *et al.*, 2016).

The maximum frame rate of the system, 1.136kHz with the current firmware, suits well the chopper repetition rate of 1kHz, allowing a full duty cycle acquisition. The integration time of the detector was set to 10us, with the integration window centered around the chopper opening time.

Dark pedestal images were collected, for all the detector gains, at the beginning of all measurement run, adding few tens of seconds to each runtime.

S8. Data processing

Using the pedestal images and per-pixel gain factors, determined in a laboratory-based dedicated calibration procedure (Redford *et al.*, 2018), the raw data were converted into photon counts and saved to multi-event HDF5 files. The initial estimate of the detector distance as well as the relative positions of two detector modules were determined from alanin powder diffraction images collected at two different detector distances.

S9. Hit-finding, indexing and integration

Diffraction images were processed with *CrystFEL* version 0.6.3 (White *et al.*, 2016), which was adapted to be able to handle non-monochromatic diffraction data. Hit finding was performed within *CrystFEL* using *peakfinder8* for peak detection and an option to skip over patterns with the number of peaks lower than a certain threshold. The images with more than 20 peaks were classified as hits. The *CrystFEL* program *indexamajig,* for indexing and integration of still diffraction images, was modified to accept the X-ray energy spectrum as an input parameter, predict Bragg reflections originating from a broader X-ray energy range and scale integrated Bragg intensities according to the intensity in the spectrum. Suppl. Fig. 2 shows a clear improvement of the Bragg spot prediction using the modified *indexamajig* version compared to the version without modifications. Suppl. Fig. 3 further reveals a significant better CC* of the datasets mainly arising from the improved spot prediction, especially at high resolution. A further slight improvement arises from scaling of the Bragg intensities according to the X-ray spectrum. The *MOSFLM*, *asdf* and *DirAx* indexing algorithms in *indexamajig* were used together with the *retry* option, which successively rejects the weakest reflections and attempts indexing until the correct indexing solution is found. The indexing results were then used to further refine detector geometry with *geoptimiser (Yefanov et al., 2015)*.

All diffraction patterns from multiple crystals (those with less than 80% predicted peaks) and patterns from crystals with high mosaicity (elongated peaks) were discarded before merging. (In total 95% of lysozyme hits and 75% of proteinaze K hits were indexed, 20% and 34% of them were discarded.) The Bragg reflection intensities from single crystal diffraction patterns were then merged with *partialator* with one round of post-refinement and without partiality correction. MTZ-files for crystallographic data processing were generated from *CrystFEL* hkl-files using *create-mtz*. Figures of merit were calculated using *compare*_hkl (R_{split}, CC*) and *check_hkl* (SNR, multiplicity, completeness), both from *CrystFEL*. The unit cell volume spatial distributions on the chip for all chips were plotted using a custom-written Python script.

S10. Structure refinements

In the case of hen-egg white lysozyme (HEWL), data were refined initially against the room-temperature model (PDB accession number: 6FTR) derived from the first successful data-collection at the European XFEL (Wiedorn *et al.*, 2018). The model was slightly modified to account for the minor differences in unit cell sizes (6FTR: a=b=79.3, c=37.73; here: a=b=79.86, c=38.04) between XFEL-data and data presented here. Initial refinement was carried out using phenix.refine (Moriarty *et al.*, 2012) and the same R_{free}-flags that were used during refinement of 6FTR. In the case of all merged patterns collected with an exposure time of 5 µs this was followed by iterative cycles of restrained maximum-likelihood refinement using phenix.refine and manual model re-building using COOT (Emsley *et al.*, 2010). The resulting model was then used for refinement of the datasets composed of a) 15000 patterns at 5 µs, b) 15000 patterns at 1 µs, c) one full chip at 5 µs. All other datasets (200, 300, 400, 500, 750, 1000, 1500, 2000, 3000, 4000, 5000, 6000, 7000, 8000 and 9000 patterns, all randomly selected subsets of the complete dataset collected at 5 µs exposure time) were just subjected to initial refinement under exactly the same conditions (same model, same refinement-parameters, same R_{free}-flags), but without manual model-rebuilding to ensure a bias-free assessment of the influence of the number of used patterns. For Proteinase K, the 5KXV structure of Proteinase K (Masuda *et al.*, 2017) was used as a starting model for molecular replacement in Phaser (McCoy *et al.*, 2007). R_{free}-flags were generated randomly using phenix.refine (Moriarty *et al.*, 2012) and the same set of R_{free}-flags were then used throughout the refinement process. Initial refinement was carried out using phenix.refine, with all isotropic atomic displacement parameters (ADP) set to 20 and using simulated annealing. This was followed by iterative cycles of restrained maximum-likelihood refinement using phenix.refine and manual model re-building using COOT (Emsley *et al.*, 2010). Polygon (Urzhumtseva *et al.*, 2009) and MolProbity (Chen *et al.*, 2010) were used for the validation of the final models.

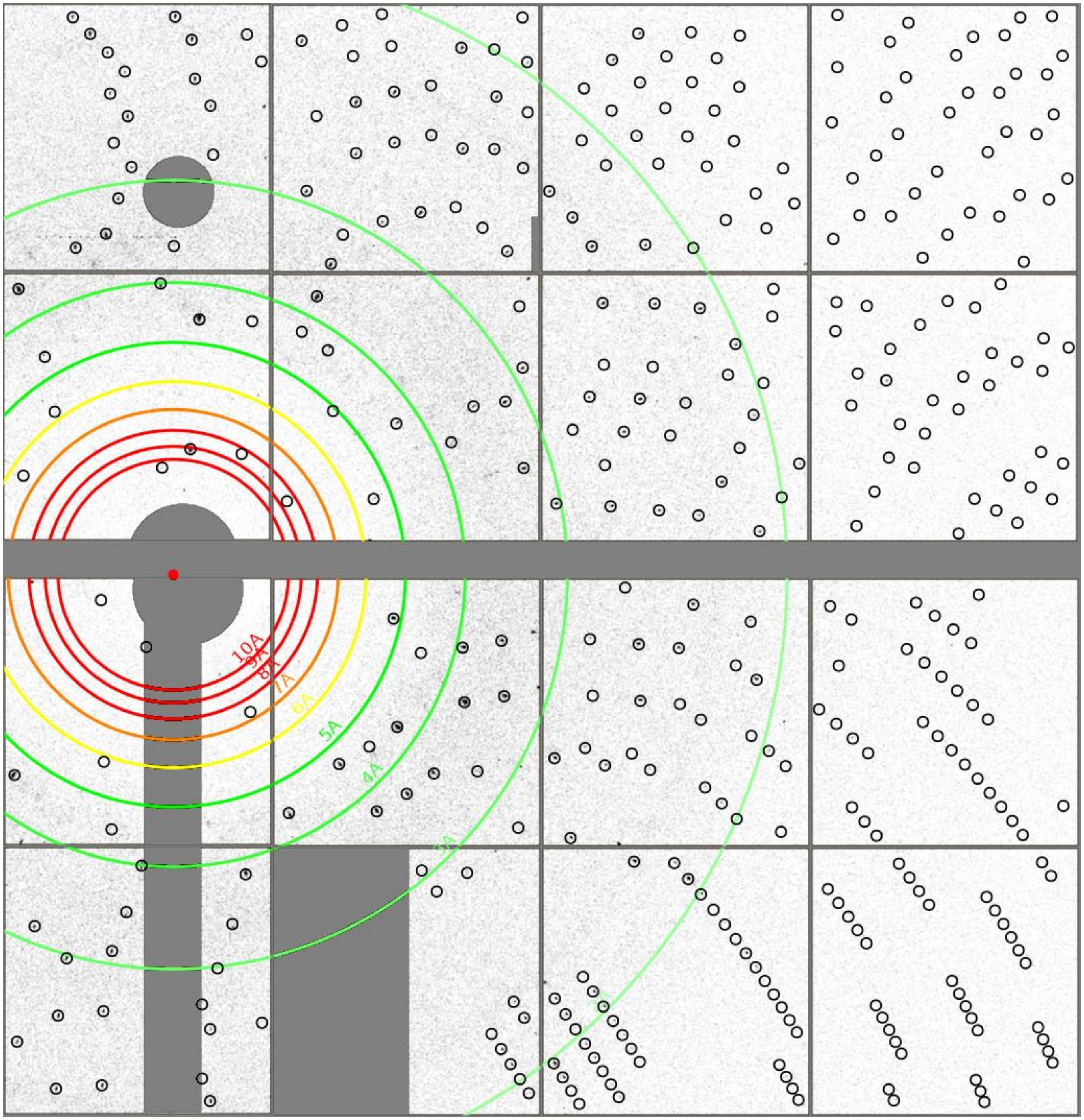

Figure S1 Lysozyme diffraction pattern indexed with *CrystFEL*, predicted Bragg spot locations shown with the black circles.

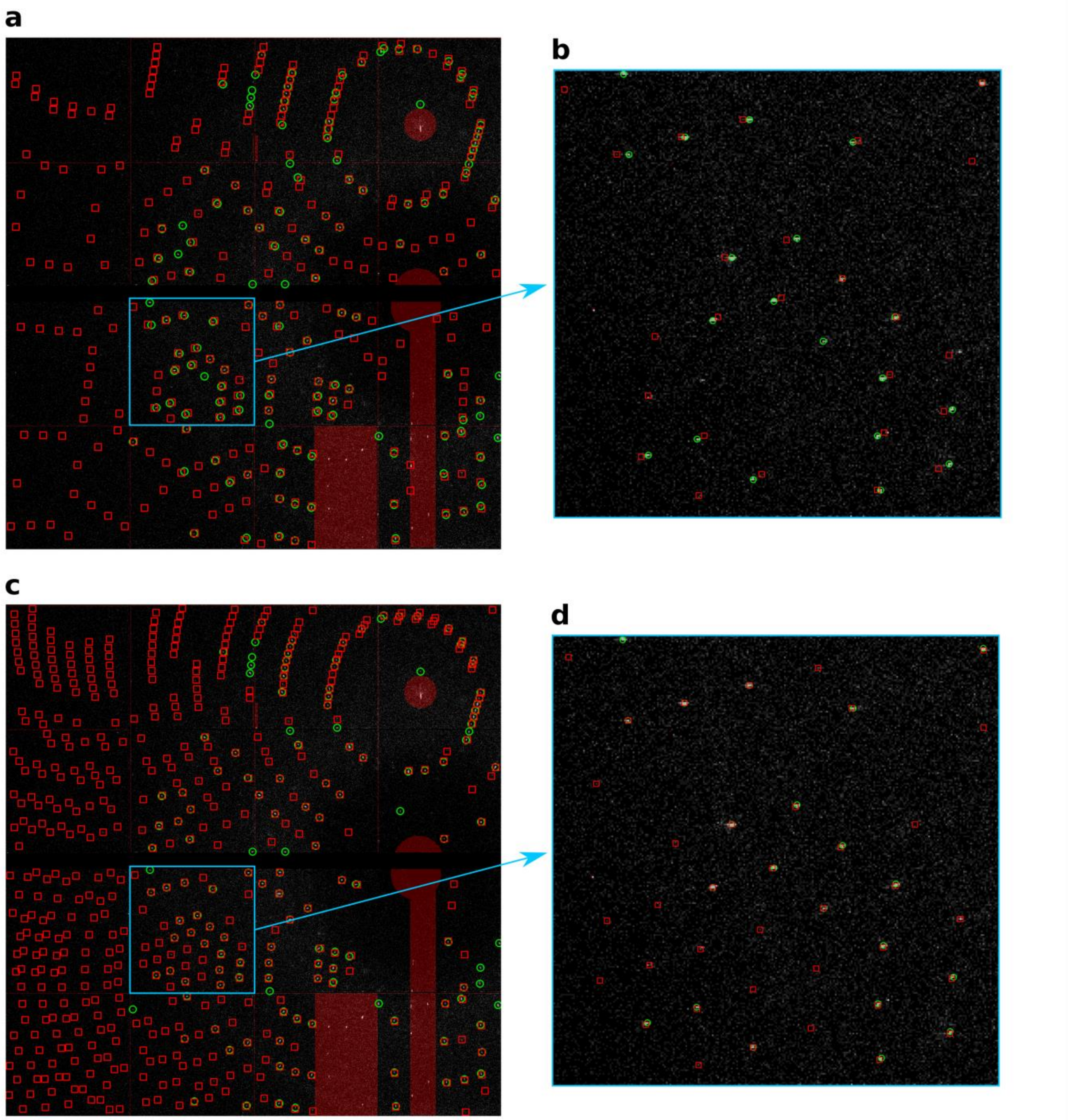

Figure S2 Lysozyme diffraction pattern indexed with *CrystFEL* without modifications (**a**) and with modifications to take into account 2.5% bandwidth (**b**). Figures (**c**) and (**d**) show magnified areas indicated in (**a**) and (**b**). Found and predicted Bragg spot positions are shown as green circles and red squares respectively.

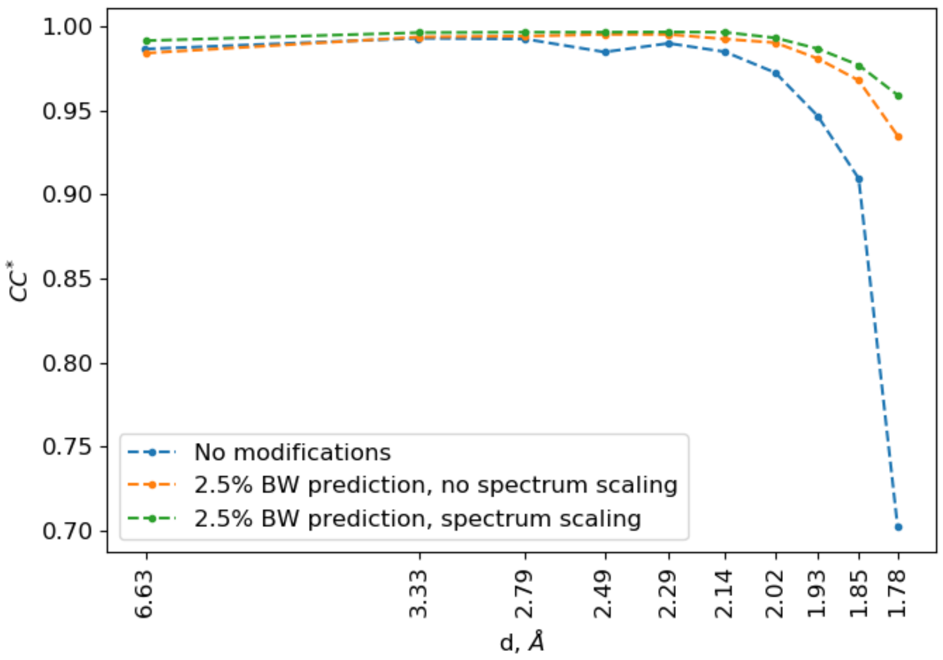

Figure S3 Comparison of *CC** as function of resolution for the dataset consisting of 8813 indexed diffraction patterns collected from the chip lys09, processed with *CrystFEL* without modifications (blue), with modifications to take into account 2.5% bandwidth (yellow) and scaling the Bragg intensities according to the intensities in the spectrum (green).

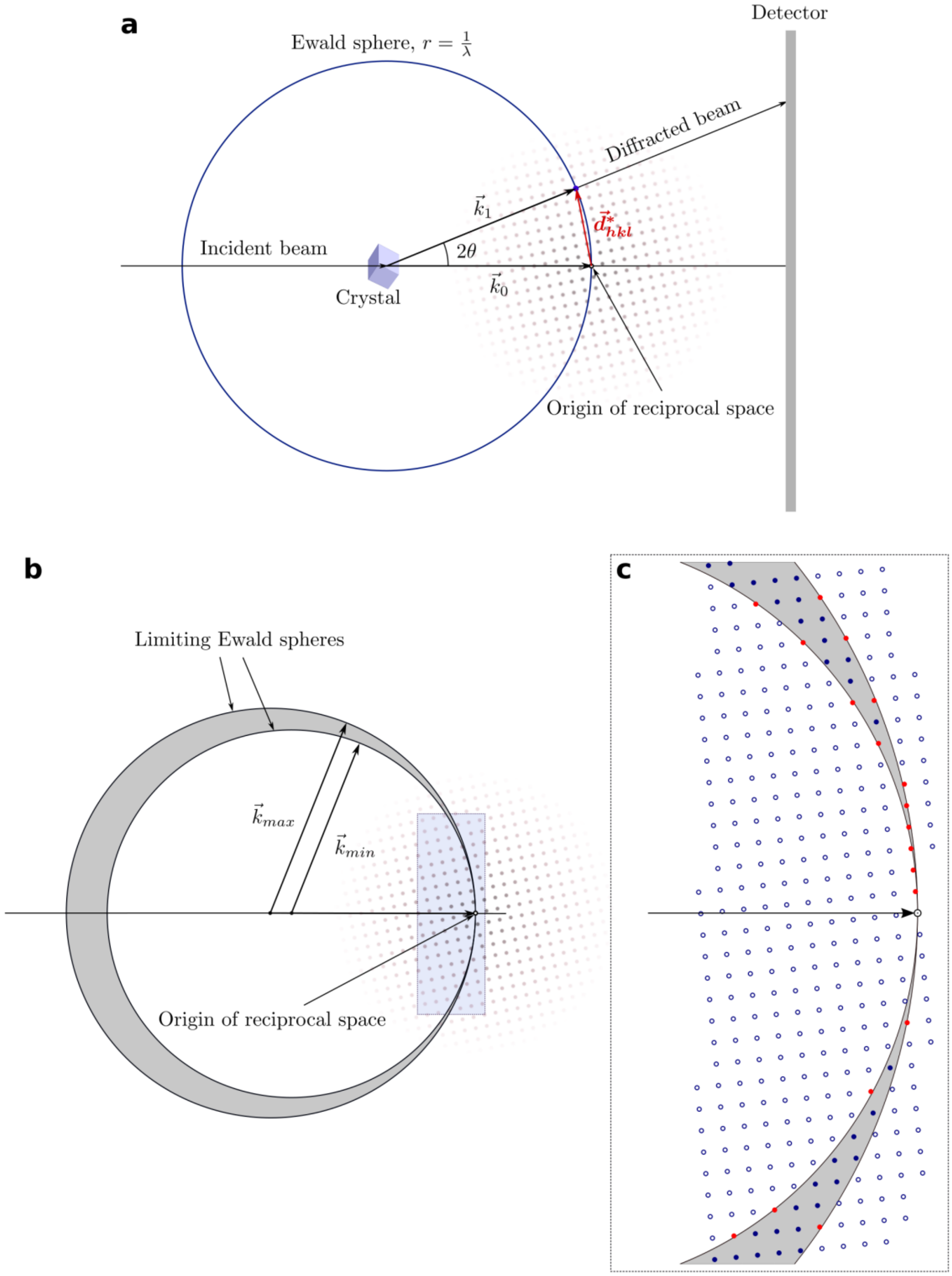

Figure S4 **a** Ewald sphere construction in case of monochromatic X-rays. Reflection will appear on the detector when a reciprocal lattice point *hkl* intersects a sphere, passing through the origin of reciprocal space with a radius of 1/λ lying along the incident beam direction, called Ewald sphere. **b** In case of the polychromatic X-rays

with a wavelengths spread between λ_{min} and λ_{max}, the Ewald sphere becomes a shell (shown in gray) between two limiting spheres with radii of $1/\lambda_{min}$ and $1/\lambda_{max}$. **c** In this case all the reflections lying fully within the shell (shown as filled blue circles) will be fully integrated. The reflections intersecting the edge of the shell and the reflections at lower resolution, where the distance between the limiting spheres is smaller than the reflection diameter (shown as red circles), will be only partially integrated. As the distance between the limiting spheres becomes larger at higher resolution the fraction of fully integrated reflections as well as the total number of diffracted reflections also increase.

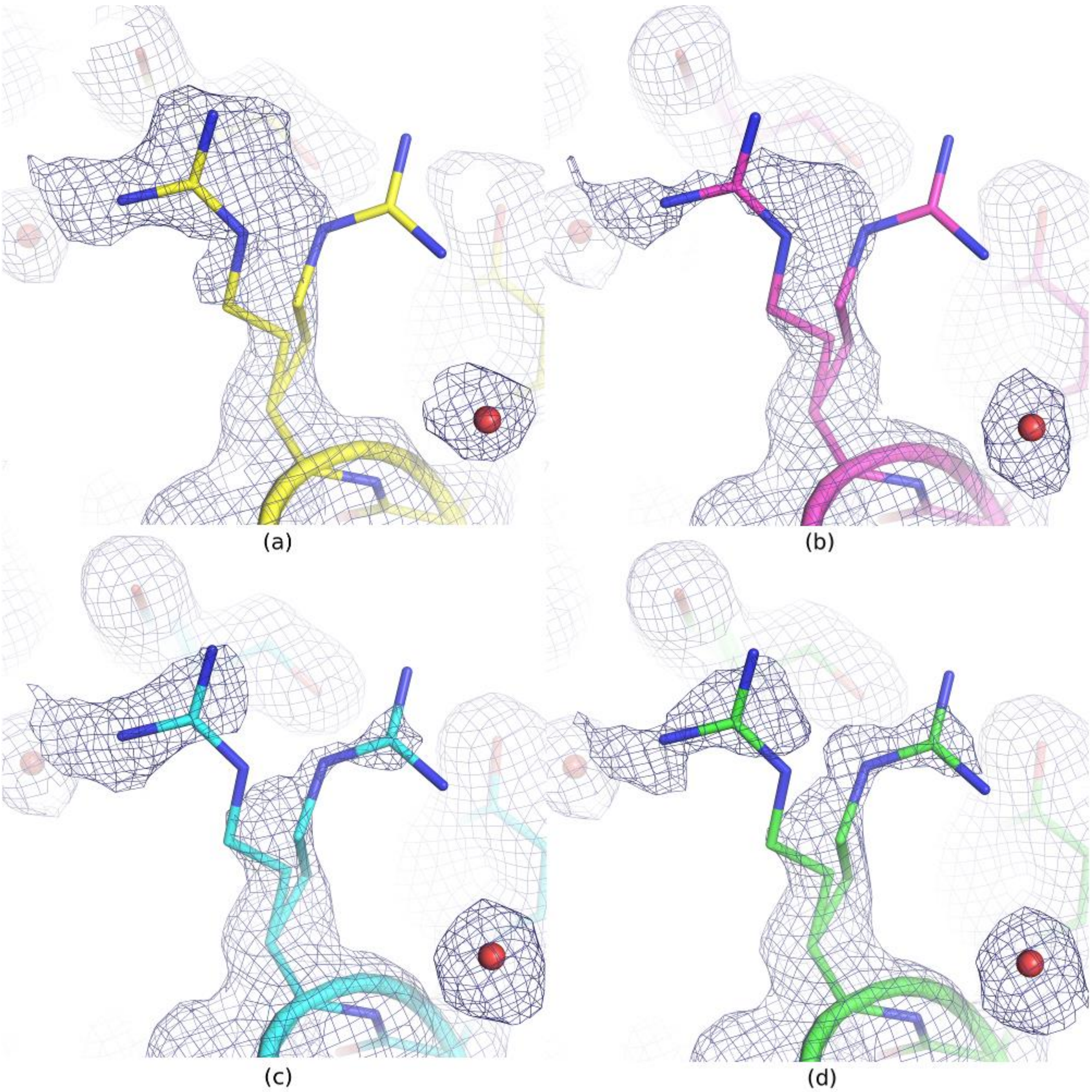

Figure S5 2mF_o-DF_c electron density maps at 0.7 sigma level of arginine 21 generated from datasets consisting of different numbers of merged diffraction patterns: (a) in yellow: 750 patterns, (b) in pink: 1500 patterns, (c) in cyan: 3000 patterns, (d) in green = all patterns (24344). The alternate conformation of this surface residue only becomes apparent when at least 3000 patterns are included in the dataset.

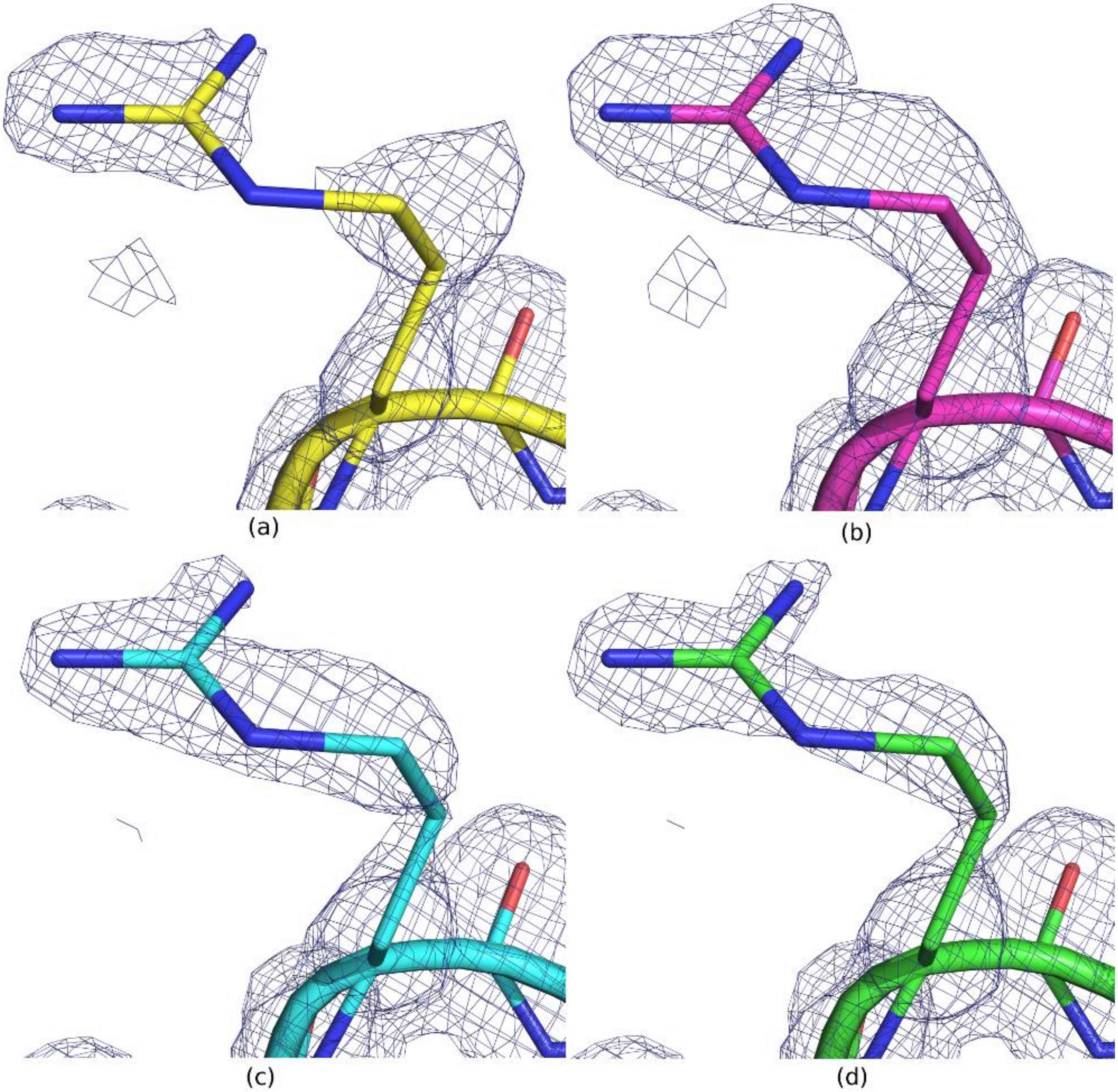

Figure S6 2mF_o-DF_c electron density maps at 0.9 sigma level around residue arginine 61 generated from datasets consisting of different numbers of merged diffraction patterns: (A) in yellow: 750 patterns, (b) in pink: 1500 patterns, (c) in cyan: 3000 patterns, (d) in green = all patterns (24344). The spacial definition of the surface residue arginine 61 improves with more patterns included in the dataset.

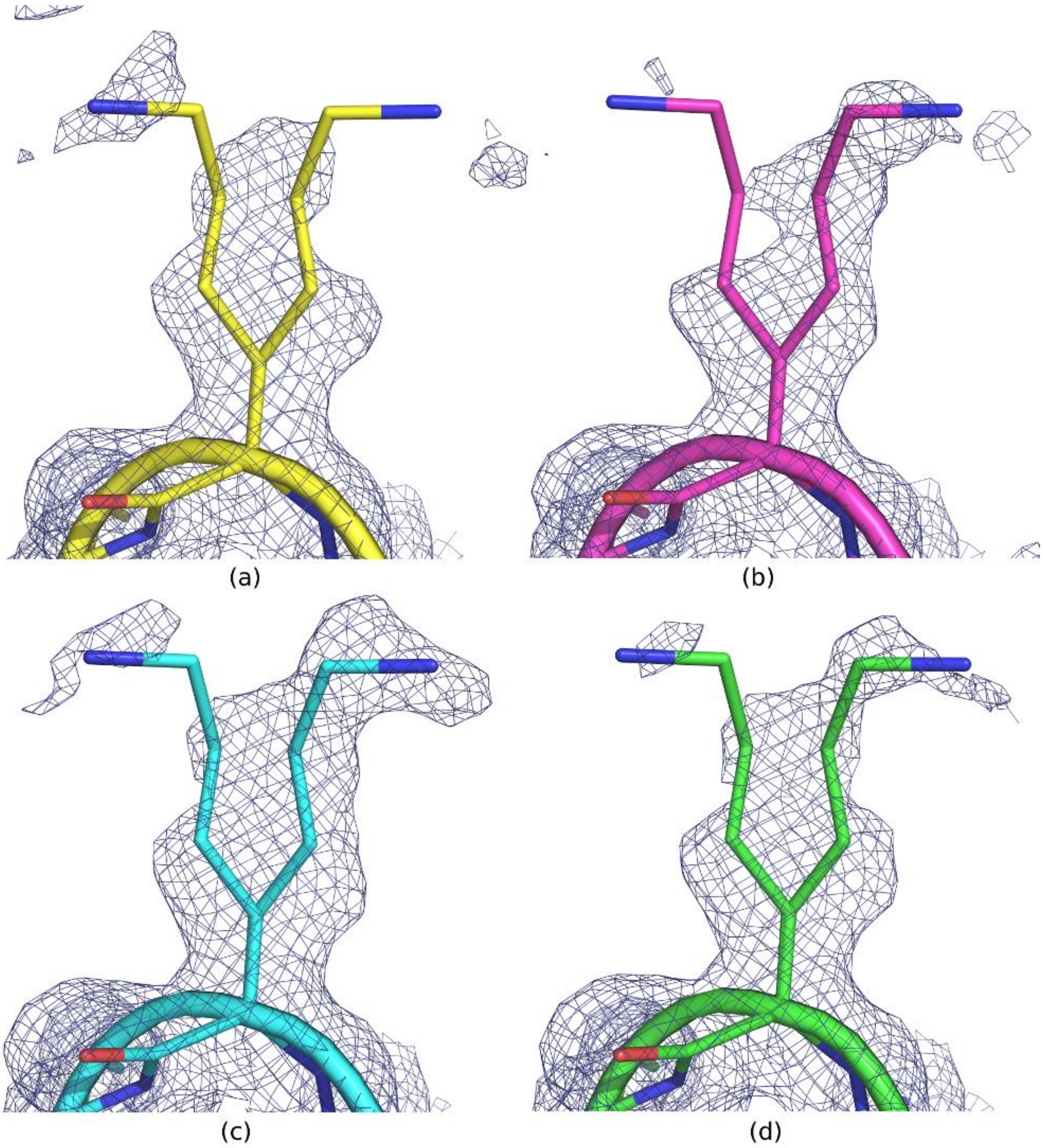

Figure S7 2mF_o-DF_c electron density maps at 0.7 sigma level of the poorly defined surface residue lysine 97 generated from datasets consisting of different numbers of merged diffraction patterns: (A) in yellow: 750 patterns, (b) in pink: 1500 patterns, (c) in cyan: 3000 patterns, (d) in green = all patterns (24344).

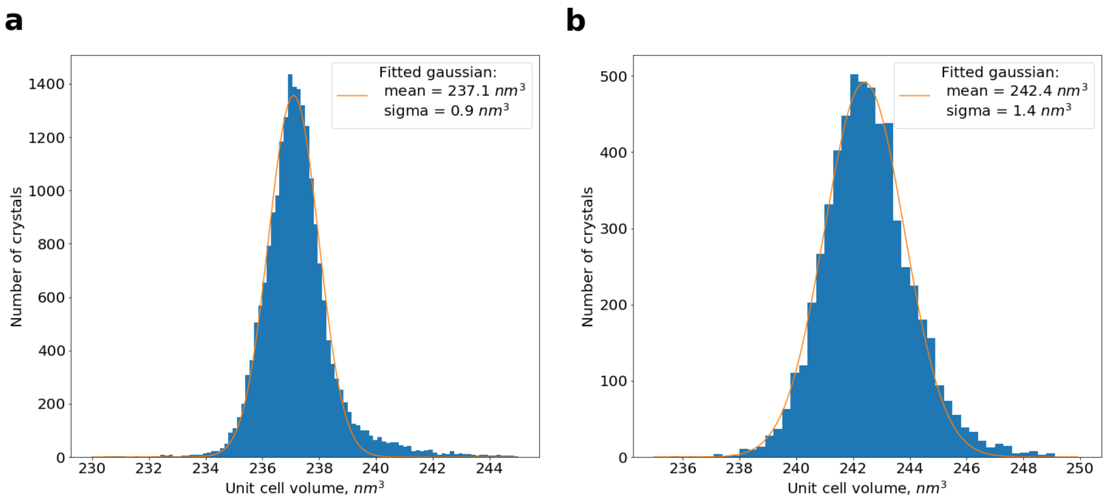

Figure S8 Unit cell volume distribution of lysozyme crystals measured in **a** EuXFEL serial crystallography experiment using jets (Wiedorn *et al.*, 2018) and in **b** experiment at beamline ID09 using fixed-target Roadrunner goniometer.

Afonine, P. V., Grosse-Kunstleve, R. W., Echols, N., Headd, J. J., Moriarty, N. W., Mustyakimov, M., Terwilliger, T. C., Urzhumtsev, A., Zwart, P. H. & Adams, P. D. (2012). *Acta Crystallogr. Sect. D Biol. Crystallogr.* **68**, 352–367.

Cammarata, M., Eybert, L., Ewald, F., Reichenbach, W., Wulff, M., Anfinrud, P., Schotte, F., Plech, A., Kong, Q., Lorenc, M., Lindenau, B., Räbiger, J. & Polachowski, S. (2009). *Rev. Sci. Instrum.* **80**, 015101.

Chen, V. B., Arendall, W. B., Headd, J. J., Keedy, D. A., Immormino, R. M., Kapral, G. J., Murray, L. W., Richardson, J. S. & Richardson, D. C. (2010). *Acta Crystallogr. Sect. D Biol. Crystallogr.* **66**, 12–21.

Emsley, P., Lohkamp, B., Scott, W. G. & Cowtan, K. (2010). *Acta Crystallogr. Sect. D Biol. Crystallogr.* **66**, 486–501.

Göries, D., Dicke, B., Roedig, P., Stübe, N., Meyer, J., Galler, A., Gawelda, W., Britz, A., Geßler, P., Sotoudi Namin, H., Beckmann, A., Schlie, M., Warmer, M., Naumova, M., Bressler, C., Rübhausen, M., Weckert, E. & Meents, A. (2016). *Rev. Sci. Instrum.* **87**, 053116.

Lieske, J., Cerv, M., Kreida, S., Komadina, D., Fischer, J., Barthelmess, M., Fischer, P., Pakendorf, T., Yefanov, O., Mariani, V., Seine, T., Ross, B. H., Crosas, E., Lorbeer, O., Burkhardt, A., Lane, T. J., Guenter, S., Bergholdt, J., Schoen, S., Toernroth-Horsefield, S., Chapman, H. N. & Meents, A. (2019). *Submitt. to IUCrJ.*

Masuda, T., Suzuki, M., Inoue, S., Song, C., Nakane, T., Nango, E., Tanaka, R., Tono, K., Joti, Y., Kameshima, T., Hatsui, T., Yabashi, M., Mikami, B., Nureki, O., Numata, K., Iwata, S. & Sugahara, M. (2017). *Sci. Rep.* **7**, 45604.

McCoy, A. J., Grosse-Kunstleve, R. W., Adams, P. D., Winn, M. D., Storoni, L. C. & Read, R. J. (2007). *J. Appl. Crystallogr.* **40**, 658–674.

Mozzanica, A., Bergamaschi, A., Brueckner, M., Cartier, S., Dinapoli, R., Greiffenberg, D., Jungmann-Smith, J., Maliakal, D., Mezza, D., Ramilli, M., Ruder, C., Schaedler, L., Schmitt, B., Shi, X. & Tinti, G. (2016). *J. Instrum.* **11**, C02047.

Redford, S., Andrä, M., Barten, R., Bergamaschi, A., Brückner, M., Dinapoli, R., Fröjdh, E., Greiffenberg, D., Lopez-Cuenca, C., Mezza, D., Mozzanica, A., Ramilli, M., Ruat, M., Ruder, C., Schmitt, B., Shi, X., Thattil, D., Tinti, G., Vetter, S. & Zhang, J. (2018). *Journal of Instrumentation*, Vol. *13*, p. C01027.

Urzhumtseva, L., Afonine, P. V., Adams, P. D. & Urzhumtsev, A. (2009). *Acta Crystallogr. Sect. D Biol. Crystallogr.* **65**, 297–300.

White, T. A., Mariani, V., Brehm, W., Yefanov, O., Barty, A., Beyerlein, K. R., Chervinskii, F., Galli, L., Gati, C., Nakane, T., Tolstikova, A., Yamashita, K., Yoon, C. H., Diederichs, K. & Chapman, H. N. (2016). *J. Appl. Crystallogr.* **49**, 680–689.

Wiedorn, M. O., Oberthür, D., Bean, R., Schubert, R., Werner, N., Abbey, B., Aepfelbacher, M., Adriano, L., Allahgholi, A., Al-Qudami, N., Andreasson, J., Aplin, S., Awel, S., Ayyer, K., Bajt, S., Barák, I., Bari, S., Bielecki, J., Botha, S., Boukhelef, D., Brehm, W., Brockhauser, S., Cheviakov, I., Coleman, M. A., Cruz-Mazo, F., Danilevski, C., Darmanin, C., Doak, R. B., Domaracky, M., Dörner, K., Du, Y., Fangohr, H., Fleckenstein, H., Frank, M., Fromme, P., Gañán-Calvo, A. M., Gevorkov, Y., Giewekemeyer, K., Ginn, H. M., Graafsma, H., Graceffa, R., Greiffenberg, D., Gumprecht, L., Göttlicher, P., Hajdu, J., Hauf, S., Heymann, M., Holmes, S., Horke, D. A., Hunter, M. S., Imlau, S., Kaukher, A., Kim, Y., Klyuev, A., Knoška, J., Kobe, B., Kuhn, M., Kupitz, C., Küpper, J., Lahey-Rudolph, J. M., Laurus, T., Le Cong, K., Letrun, R., Xavier, P. L., Maia, L., Maia, F. R. N. C., Mariani, V., Messerschmidt, M., Metz, M., Mezza, D., Michelat, T., Mills, G., Monteiro, D. C. F., Morgan, A., Mühlig, K., Munke, A., Münnich, A., Nette, J., Nugent, K. A., Nuguid, T., Orville, A. M., Pandey, S., Pena, G., Villanueva-Perez, P., Poehlsen, J., Previtali, G., Redecke, L., Riekehr, W. M., Rohde, H., Round, A., Safenreiter, T., Sarrou, I., Sato, T., Schmidt, M., Schmitt, B., Schönherr, R., Schulz, J., Sellberg, J. A., Seibert, M. M., Seuring, C., Shelby, M. L., Shoeman, R. L., Sikorski, M., Silenzi, A., Stan, C. A., Shi, X., Stern, S., Sztuk-Dambietz, J., Szuba, J., Tolstikova, A., Trebbin, M., Trunk, U., Vagovic, P., Ve, T., Weinhausen, B., White, T. A., Wrona, K., Xu, C., Yefanov, O., Zatsepin, N., Zhang, J., Perbandt, M., Mancuso, A. P., Betzel, C., Chapman, H. & Barty, A. (2018). *Nat. Commun.* **9**, 4025.

Yefanov, O., Mariani, V., Gati, C., White, T. A., Chapman, H. N. & Barty, A. (2015). *Opt. Express*. **23**, 28459.
